# Click-Ready Gold Nanoparticles from Aqueous Mechanochemistry: 2-Propynylamine as a Reducing Agent and Surface Ligand

**DOI:** 10.3390/ma18194470

**Published:** 2025-09-25

**Authors:** Amber L. Garcia, Brian S. Mitchell, Amanda Reusch, Mark J. Fink, Juan P. Hinestroza, Yelin Ko, Julie P. Vanegas

**Affiliations:** 1 College of Engineering and Computer Science, The University of Texas Rio Grande Valley, Edinburg, TX 78539, USA; amber.garcia05@utrgv.edu; 2 Department of Chemical and Biomolecular Engineering, Tulane University, New Orleans, LA 70118, USA; brian@tulane.edu (B.S.M.); areusch@tulane.edu (A.R.); 3 Department of Chemistry, Tulane University, New Orleans, LA 70118, USA; fink@tulane.edu; 4 Department of Human Centered Design, College of Human Ecology, Cornell University, Ithaca, NY 14850, USA; jh433@cornell.edu (J.P.H.); yk563@cornell.edu (Y.K.); 5 Department of Physics and Astronomy, The University of Texas Rio Grande Valley, Edinburg, TX 78539, USA

**Keywords:** gold nanoparticles (AuNPs), bifunctional reducing ligand, 2-propynylamine, reactive high-energy ball milling (RHEBM), terminal alkyne functionalization, mechanocatalytic approach, click-ready nanomaterials, bioconjugation

## Abstract

We report a rapid aqueous method for synthesizing monodisperse gold nanoparticles (AuNPs), employing 2-propynylamine as both an intrinsic reducing agent and a surface-stabilizing ligand. This self-mediated process—achieved in a single step—yields spherical AuNPs with an average diameter of 4.0 ± 1.0 nm and a well-defined localized surface plasmon resonance band centered at 520 nm. Acting as a bifunctional molecule, 2-propynylamine simultaneously reduces HAuCl_4_·3H_2_O to elemental gold and passivates the nanoparticle surface through coordination via the amine group, while preserving a terminal alkyne (–C≡CH) functionality. This reactive moiety remains exposed and chemically accessible, enabling post-synthetic modification through Cu(I)-catalyzed azide–alkyne cycloaddition. Control experiments using alternate milling times and vial composition confirmed the essential role of 2-propynylamine in mediating both reduction and surface functionalization. The resulting alkyne-functionalized AuNPs serve as versatile “click-ready” platforms for bioconjugation, sensing, and advanced material assembly. Overall, this scalable, green approach eliminates the need for external reducing or capping agents and provides a modular route to chemically addressable nanomaterials with tunable surface reactivity.

## 1. Introduction

Gold nanoparticles (AuNPs) are widely used in catalysis, sensing, electronics, and biomedicine due to their tunable optical properties, biocompatibility, and high surface-to-volume ratio. However, conventional synthesis methods typically require external reducing agents and stabilizers, which can prolong reaction times, generate chemical waste, and complicate surface modification. While recent approaches—such as controlling the phosphate-to-HEPES molar ratio [1,2], microwave-assisted synthesis [3], and citrate reduction [4]—have reduced synthesis times to under 15 min, they still rely on multicomponent systems and post-functionalization steps. These limitations hinder the direct integration of AuNPs into advanced applications where precise surface chemistry is essential, such as in biosensors, drug delivery systems, and click-mediated bioconjugation [5,6].

All these protocols still rely on the presence of explicit reducing agents—such as citrate, HEPES (4-(2-hydroxyethyl)-1-piperazineethanesulfonic acid) or sodium borohydride—to drive the reduction of gold ions into nanoparticles [4,5,6]. Newer approaches, like the use of ligands as both a reducing agent and stabilizer, eliminate the need for separate reducing reagents, simplifying the synthesis process. These advancements represent an ongoing evolution in nanoparticle synthesis techniques, not only reducing synthesis time but also minimizing the complexity of reagent use, enhancing the scalability and functionality of nanoparticle synthesis.

Bottom-up synthesis techniques, particularly those utilizing the mechanochemical reduction of gold salts in a sealed container using a ball milling device, have emerged as promising alternatives to conventional wet-chemical synthesis methods for metallic nanoparticles [7,8,9]. Traditional solution-based approaches—such as the Turkevich and Frens methods—generally require solvents, external reducing agents, and additional stabilizers, which can complicate the process and generate chemical waste [10]. In contrast, mechanochemical synthesis, especially when combined with primary amines as reducing agents, has been successfully applied to the reduction in various metals, including manganese, iron, gold, copper, vanadium, platinum, and bimetallic Pt/Ir systems [11,12,13,14]. This aqueous methodology offers several key advantages over traditional techniques, such as eliminating the need for solvents, reducing reaction times, and can lead to higher yields. The mechanical activation inherent in this process enables more efficient, sustainable chemical reactions, positioning mechanochemistry as a viable method for synthesizing a broad range of materials [7,9].

Pioneering work in this field has achieved controlled AuNP particle sizes by reducing KAuCl_4_ with NaBH_4_ in the presence of polyvinylpyrrolidone (PVP) [15,16]. A mechanochemical approach has also been used to synthesize gram-scale quantities of amine-capped AuNPs with uniform sizes of approximately 1–4 nm [8]. The latter method eliminated the need for external reducing agents or solvents, as the steel milling assembly itself acts as a galvanic reducing agent. While prior investigations have primarily focused on primary amines, this study extends this methodology to bifunctional amines, which has not been reported in the literature before.

This method for the synthesis of 2-propynylamine-passivated AuNPs can be easily adapted to other bifunctional ligands, including thiols, carboxyl, and amines. These terminal reactive groups create functionalized AuNPs that are appropriate for click chemistry (e.g., with azides and alkynes), which provides a precise and efficient route to perform coupling reactions, such as [2 + 3] cycloadditions [17,18,19]. These click reactions are known for their reliability and selectivity under mild conditions. This adaptability is especially valuable for bioconjugation processes, where the stability and versatility of the reaction conditions are paramount.

This research presents a comprehensive investigation into the synthesis of AuNPs via reactive high-energy ball milling (RHEBM), using 2-propynylamine as a dual-function ligand that serves both as an intrinsic reducing agent and as a surface stabilizer. The resulting AuNPs were thoroughly characterized using UV–Vis spectroscopy, X-ray diffraction (XRD), X-ray photoelectron spectroscopy (XPS), and analyses of surface-ligand interactions via nuclear magnetic resonance spectroscopy (NMR), thermogravimetric analysis (TGA), and Fourier-transform infrared spectroscopy (FTIR). Beyond demonstrating a scalable, and rapid synthesis route, this study emphasizes the unique surface functionality imparted by the terminal alkyne group (–C≡CH) of 2-propynylamine, which remains chemically accessible for post-synthetic modification. This key feature enables biorthogonal conjugation via Cu(I)-catalyzed azide–alkyne cycloaddition (CuAAC), making the resulting nanoparticles readily adaptable for applications in biosensing, drug delivery, and nanomaterial assembly [20,21].

While the RHEBM method relies on the input of high mechanical energy, it substantially reduces or eliminates the use of hazardous chemical reagents and solvents compared to conventional solution-based syntheses. As a result, this technique not only aligns with the principles of green chemistry but also provides a sustainable and versatile platform for the synthesis of chemically addressable nanomaterials that can be readily tailored for advanced technological and biomedical applications.

## 2. Materials and Methods

### 2.1. Materials

All chemicals were analytical grade and were used without further purification. 2-Propynylamine (HC≡CCH_2_NH_2_, 98%) was purchased from Alfa Aesar (Ward Hill, MA, USA), chloroauric acid trihydrate (HAuCl_4_∙3H_2_O, 99.9%) and iron flakes (Fe, ≥99.99%, trace metals basis) were purchased from Sigma-Aldrich (St. Louis, MO, USA). Deionized (DI) water was purified with a Milli-Q Direct-Q^®^ 3 UV Water Purification System (Merck Millipore, Burlington, MA, USA).

### 2.2. Methods

#### 2.2.1. Synthesis of AuNPs Passivated with 2-Propynylamine

0.508 mmol of chloroauric acid trihydrate (HAuCl_4_·3H_2_O) and 1.632 mmol of 2-propynylamine (C_3_H_5_N)—corresponding to an approximate 1:3 molar ratio of gold to ligand—were added to 30 mL of DI water. The mixture was placed into a hardened stainless-steel milling vial along with two stainless-steel milling balls, each 5 mm in diameter and weighing 0.21 g.

The vial contents were subjected to RHEBM using a SPEX CertiPrep 8000D Mixer/Mill (SPEX SamplePrep, Metuchen, NJ, USA) for 2.5 h in a cold room around 4 °C to prevent overheating and maintain stable conditions during the high-energy process. After milling, the balls were removed, and the vial contents were centrifuged at 3800 rpm for 30 min in sterile, flat-capped, 50 mL conical-bottomed plastic tubes (VWR brand), separating the mixture into liquid (supernatant) and solid (pellet) fractions.

The pellet was dried for 3 h at 85 °C to remove any unreacted precursor, then weighed. This material was designated as AuNPs passivated with 2-propynylamine (Au@2-propynylamine NPs). The pellet readily dispersed in DI water, yielding a burgundy-colored solution. The original supernatant produced a clear, colorless solution after evaporation and re-dispersion in DI water. This step was carried out to remove excess unbound 2-propynylamine ligand and to confirm that no residual gold nanoparticles remained in the supernatant, as evidenced by the absence of the characteristic burgundy color.

#### 2.2.2. Control Experiments: The Role of RHEBM and Reflux in Nanoparticle Formation

A series of eight control experiments, along with one reflux experiment, were conducted to elucidate the influence of RHEBM on nanoparticle formation. These experiments aimed to replicate the conditions of the RHEBM process without the milling action by varying the type of vial used or adding iron flakes to replicate the environmental conditions without subjecting the mixture to ball milling.

Each control experiment followed the following procedure: A mixture of HAuCl_4_·3H_2_O and 2-propynylamine was prepared in DI water at room temperature to evaluate whether any variations would produce a burgundy-colored solution, indicative of plasmonic nanoparticle formation. The vials were covered with aluminum foil to shield the reaction from light and stirred continuously overnight. After completion, each reaction was subjected to a series of analyses, including UV-Vis spectroscopy to detect the characteristic surface plasmon resonance band (typically 520–550 nm) associated with gold nanoparticles, as well as visual inspection and photographic documentation of color changes. In selected cases, transmission electron microscopy (TEM) was performed to confirm the presence, size, and morphology of nanoparticles, and dynamic light scattering (DLS) was used to assess hydrodynamic diameter and particle size distribution. Additionally, reactions were evaluated using glass, methacrylate, or zirconia vials in place of steel vials, and with the addition of iron flakes, to determine whether the material of the container or the presence of iron influenced the development of plasmonic nanoparticles. The control experiments were as follows:

- Zirconia milling vial + stirring (Vial F);
- Zirconia milling vial + iron flakes + stirring (Vial A);
- Zirconia milling vial + ball milling (Vial H);
- Steel milling vial + iron flakes + stirring (Vial E);
- Steel milling vial + ball milling (Vial G);
- Methacrylate milling vial + stirring (Vial C);
- Glass storage vial + stirring (Vial B);
- Glass storage vial + iron flakes + stirring (Vial D);
- Reflux: The same reaction conditions as RHEMB, evaluating the time it takes for the solution to change from yellow to burgundy.

The control experiments were designed to isolate the specific contributions of mechanical energy, container material, and metal presence to nanoparticle synthesis. Comparing the outcomes of these controls with those from RHEBM-processed samples yielded insights into the role of mechanical processes in nanoparticle formation through RHEBM.

#### 2.2.3. Time-Control Experiments

RHEBM synthesis was conducted in stainless steel vials with reaction durations of 15, 30, 45, 60, 90, and 120 min to determine whether Au@2-propynylamine NPs could be effectively synthesized in a shorter timeframe.

#### 2.2.4. Characterization of Gold Nanoparticles and Related Materials

Ultraviolet–visible (UV-Vis) measurements were conducted using a Varian Cary 50 UV-Vis spectrophotometer (Varian, Inc., Palo Alto, CA, USA). Samples were placed in 1 cm quartz cuvettes at room temperature, and spectra were obtained in the range of 200–800 nm. A constant-temperature sipper system was used for kinetic measurement and absorption spectra. UV-Vis spectral data were processed using OriginPro 2023 software [22].

Proton Nuclear Magnetic Resonance (^1^H-NMR) and Carbon-13 Nuclear Magnetic Resonance (^13^C-NMR) spectroscopy, and distortionless enhancement by polarization transfer (DEPT-135) measurements were recorded using a Bruker DSX 300 multinuclear NMR spectrometer (Bruker, Billerica, MA, USA). The NMR samples were prepared using deuterated dimethylsulfoxide (DMSO-d_6_; 99.9 atom% D) or deuterium oxide (D_2_O; 99.9 atom% D) as a solvent, which were both obtained from Sigma-Aldrich. All 1D and 2D NMR spectral data were processed using MestReNova software 10.1.0-16742 [23].

X-ray Photoelectron Spectroscopy (XPS) spectra were collected with a ThermoFisher Scientific Nexsa G2 Surface Analysis System (ThermoFisher, Waltham, MA, USA) using a focused Al Kα monochromatic X-ray source (1486.6 eV). Samples were deposited on an indium foil (5 mm × 5 mm, 4 mm thick) via drop casting from an aqueous dispersion, followed by drying under ambient conditions. Spectral peak fitting and deconvolution were performed with CasaXPS software 2.3.25PR1.0 [24], using Gaussian-Lorentzian line shapes.

Transmission electron microscopy (TEM) images were obtained using a JEOL 2010 TEM (JEOL, Tokyo, Japan), with a 300 kV accelerating voltage and a 0.23 nm point resolution. Samples were prepared by dipping standard carbon-coated (200–300 Å), Formvar-covered copper grids (200-mesh) into a dilute solution of AuNPs in water or methanol. TEM grids were then allowed to dry under ambient conditions for 24 h. The size distributions of the AuNPs were determined from the diameters of at least 500 particles located in a representative region of the micrographs using ImageJ software 1.53k [25].

Fourier Transform Infrared (FTIR) spectroscopy was performed using a Nicolet iS50 attenuated total reflectance (ATR) module (Thermo Fisher Scientific, Waltham, MA, USA) with a diamond crystal. Samples were smeared using a spatula to ensure full coverage of the crystal. Scans were taken between 4000 and 400 cm^−1^ at a resolution of 4 cm^−1^. All resulting spectra were plotted in OriginPro 2023 [22].

X-ray diffraction (XRD) analysis was performed using a Rigaku diffractometer (Rigaku, Tokyo, Japan) equipped with a Cu Kα radiation source (λ = 1.5406 Å). The samples—dried gold nanoparticles (AuNPs) obtained from mechanochemical synthesis, solution-based controls, and any relevant intermediate solid products—were prepared by grinding the materials into a fine powder that was evenly spread onto a sample holder. The measurements were conducted in the 2θ range from 10° to 80°, with a step size of 0.02° and a counting time of 1 s per step. The voltage and current of the X-ray source were set to 40 kV and 30 mA, respectively. All resulting diffraction patterns were analyzed and plotted using OriginPro 2023 software [22].

#### 2.2.5. Surface Alkyne Post-Synthetic Click Functionalization with Biotin-PEG_4_-Azide and Controls

To demonstrate the chemical accessibility and reactivity of the terminal alkyne groups on Au@2-propynylamine nanoparticles, Cu(I)-catalyzed azide–alkyne cycloaddition (CuAAC) was performed using Biotin-PEG_4_-azide as a prototypical ligand [26,27]. In this approach, the biotin moiety serves as a high-affinity biomolecular recognition element, while the PEG_4_ spacer and terminal azide enable efficient, site-specific covalent attachment of the Biotin-PEG_4_ ligand to the nanoparticle surface through triazole formation [28,29]. This strategy ensures that the resulting gold nanoparticles are functionalized with biotin groups, making them suitable for further bioconjugation and recognition-based applications.

A total of 30 mg of Au@2-propynylamine nanoparticles were dispersed in 10.0 mL of ultrapure water to achieve a final concentration of 3.0 mg/mL. Separately, biotin-PEG_4_-azide was dissolved at 15 mM in DMSO-d_6_ or ethanol; 20 µL of this stock solution was added to the nanoparticle suspension, resulting in a final azide concentration of approximately 30 µM. For the CuAAC click reaction, 25 µL of 40 mM CuSO_4_·5H_2_O and 90 µL of freshly prepared 56 mM sodium ascorbate were sequentially introduced, generating catalytically active Cu(I) in situ. The mixture was stirred at room temperature (22–25 °C) for 6 h and protected from light to prevent photodegradation of the reagents. After incubation, the reaction mixture was centrifuged at 10,000 rpm for 15 min, and the pellet was washed three times with ultrapure water to remove excess reagents and byproducts. The final product was redispersed in either ultrapure water or 1× phosphate-buffered saline (PBS) to assess colloidal stability under physiological conditions.

To confirm the specificity of the click reaction and exclude nonspecific interactions, the following controls were conducted in parallel:

Control C1: AuNPs were suspended in 1× PBS to assess how ionic strength affects the colloidal behavior of the nanoparticles.

Control C2: AuNPs were incubated with Biotin-PEG_4_-azide in the absence of both copper sulfate and ascorbate. This control was critical for evaluating the potential for nonspecific adsorption of the azide ligand onto the nanoparticle surface.

Control C3: AuNPs were treated with copper sulfate and ascorbate, without the azide ligand. This condition was designed to assess the impact of in situ-generated Cu(I) ions on nanoparticle stability.

Control C4: The control contained Biotin-PEG_4_-azide, copper sulfate, and ascorbate, without any AuNPs.

#### 2.2.6. Nanoparticle-ELISA Validation with Streptavidin-HRP and TMB

A nanoparticle-based ELISA (NP-ELISA) was performed using streptavidin-HRP and 3,3′,5,5′-tetramethylbenzidine (TMB) as the chromogenic substrate [30,31] to validate the presence and accessibility of biotin on the surface of AuNPs after click functionalization. Briefly, biotinylated AuNPs were resuspended in PBS at a concentration of 0.2–0.3 mg/mL and incubated with streptavidin-HRP (1:1000 dilution in PBS) for 30–60 min at room temperature, protected from light. After incubation, any unbound enzyme was removed by centrifugation (for tubes) or repeated PBS washes (for plates), and the nanoparticle pellet was resuspended in PBS. Subsequently, 100 µL of TMB substrate—either as a ready-to-use solution or freshly prepared by dissolving TMB powder in citrate buffer with 0.03% H_2_O_2_—was added to each sample and incubated for 10–15 min in the dark. The development of a blue color indicated HRP activity and, consequently, the presence of accessible biotin on the nanoparticle surface [32].

## 3. Results and Discussion

**Figure 1:**
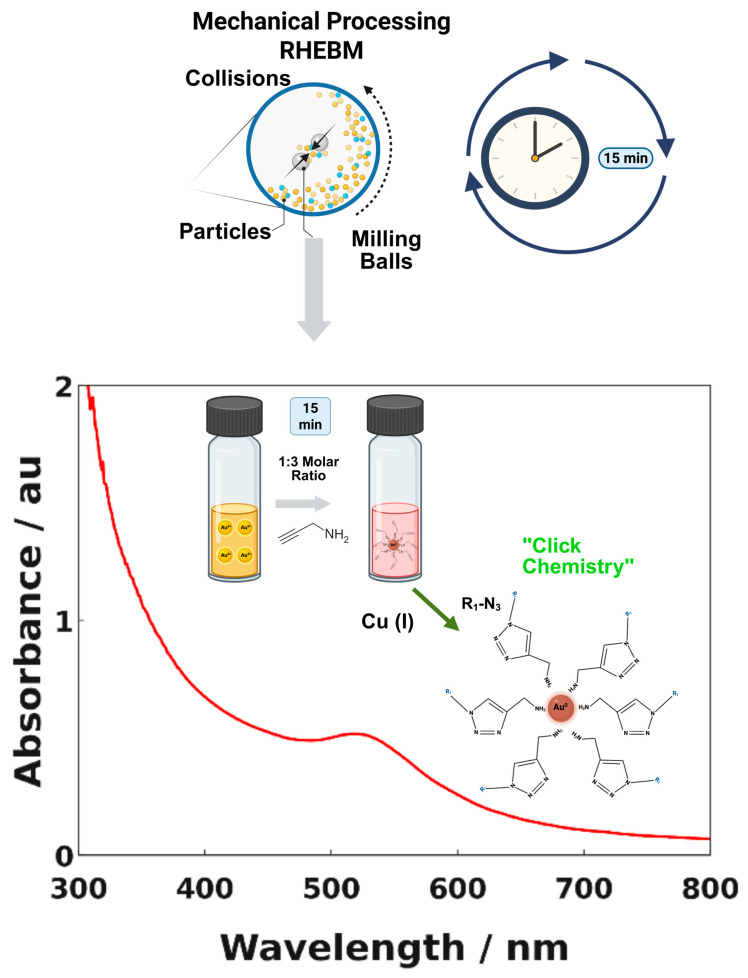
Schematic Illustration of the Synthesis of Au@2-propynylamine NPs and UV-Vis Spectra in Water.

The material obtained from the stainless-steel milling vial (see Section 2.2.1) after 2.5 h of RHEBM exhibited a notable color change, from the yellow hue in the precursor solution (typical for gold salt solutions) to a deep, burgundy aqueous suspension of the pellet. This color change indicates the successful reduction of gold ions to form AuNPs. UV-Vis measurements, shown in Figure 1, further validated this synthesis, with the pellet solution displaying a pronounced absorption band centered around 520 nm—a signature feature of plasmonic nanoparticles [33,34,35]. Optimization of the Au:2-propynylamine molar ratio significantly influenced the synthesis yield, with triplicate syntheses yielding 60.0 ± 15.0% for 1:10, 54.7 ± 19.6% for 1:5, and 87.7 ± 6.6% for 1:3, the latter offering the highest reproducibility and efficiency (Table S1, Supporting Information).

In contrast, the supernatant showed no absorbance near 520 nm, indicating the absence of AuNPs. In the Supporting Information (Figures S1–S3), comprehensive characterization of the supernatant by ^1^H and ^13^C NMR, UV-Vis spectroscopy, and FTIR confirms that only excess ligand in water is present. Time control experiments indicated that nanoparticle synthesis can be achieved under these conditions with just 15 min of exposure to ball milling and the primary amine (refer to Supporting Information, Figures S4 and S5).

To better understand the influence of reaction time, a detailed comparison of the time-controlled experiments (15, 30, 45, 60, 90, 120, and 150 min) was carried out (Figure S5, Supporting Information). The localized surface plasmon resonance (LSPR) band at ~320 nm was already visible after 15 min, indicating the onset of AuNP formation. Between 30 and 60 min, the LSPR band became progressively more intense and slightly red-shifted, consistent with nanoparticle growth and improved crystallinity. At 90 and 120 min, the LSPR intensity approached a plateau, suggesting that most gold ions had been reduced and incorporated into nanoparticles. At 150 min, the reaction reached completion, as evidenced by the stable plasmon band position and maximum intensity, with no further spectral changes observed.

**Figure 2:**
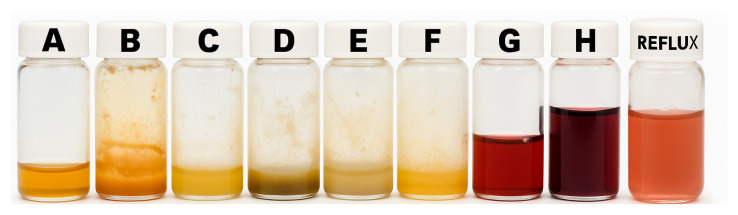
Control Samples. Vials (**A**–**F**) show no coloration. Vials (**G**,**H**) and the reflux sample exhibit a burgundy-colored solution, indicating the presence of plasmonic AuNPs.

Figure 2 shows the samples produced under the various synthesis conditions outlined in Section 2.2.2. RHEBM of samples in the steel vial (Figure 2G) and the zirconia vial (Figure 2H) produced burgundy-colored solutions indicative of AuNP formation. Samples that were stirred without RHEMB showed no evident change with or without iron flakes. Additionally, a color change and the development of plasmonic nanoparticles were observed after 3 h of reflux, indicating that RHEBM and reflux can achieve the desired synthesis through different mechanisms and efficiencies (See Supporting Information, Figures S6 and S7).

We postulate that AuNPs are formed only in milling or reflux conditions due to energy considerations. The high-energy mechanical forces in RHEBM significantly accelerate the reaction, leading to faster nanoparticle formation, unlike the slower kinetics in reflux, which rely solely on thermal energy [36,37]. RHEBM provides continuous and intense energy input through mechanical collisions, quickly breaking chemical bonds and creating reactive sites. In contrast, the heat energy of reflux is localized and less intense, resulting in slower bond-breaking and formation processes [38,39]. The mechanical action of RHEBM can create numerous nucleation sites, promoting faster nanoparticle growth. The localized high temperatures and pressures of RHEBM drive reactions not feasible under normal reflux conditions, which typically occur at the solvent’s boiling point, with relatively lower temperatures and pressures than the localized conditions in RHEBM [40,41].

### 3.1. Transmission Electron Microscopy

**Figure 3:**
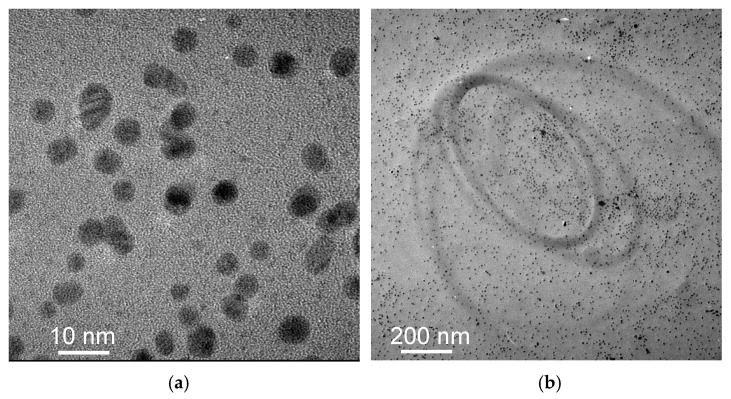
(**a**) TEM image of Au@2-propynylamine NPs at 10 nm scale; (**b**) TEM image at 200 nm scale.

Figure 3a,b are TEM images of Au@2-propynylamine NPs milled in stainless steel vials, showing the spherical morphology of AuNPs. The size distribution of the dispersed AuNPs in water is roughly monomodal, with particle sizes primarily between 3 and 5 nm and an average diameter of 4.0 ± 1.0 nm.

Panels (a) and (b) provide compelling evidence for the high degree of homogeneity in the synthesized nanoparticles, both in terms of size and spatial distribution. The TEM image in panel (a) reveals nanoparticles with a remarkably uniform average diameter of 4.0 ± 1.0 nm, with minimal size dispersion and no significant aggregation. The lower-magnification TEM image in panel (b) shows an even spatial distribution of nanoparticles over a larger area, supporting the conclusion that the sample is morphologically homogeneous at the nanoscale.

### 3.2. X-Ray Diffraction

**Figure 4:**
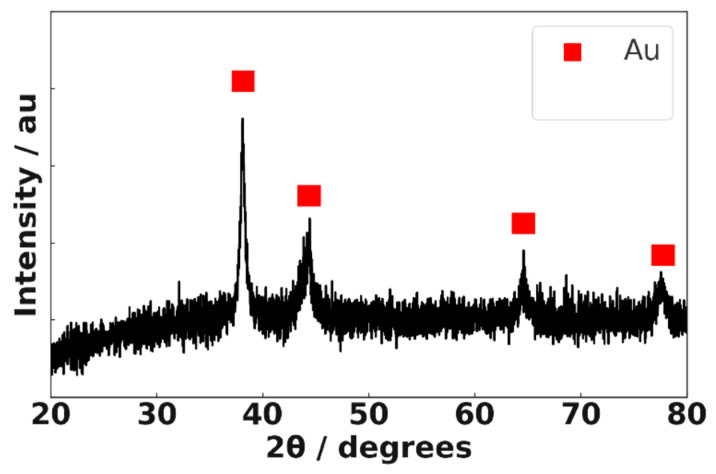
XRD pattern of Au@2-propynylamine NPs. The prominent diffraction peaks (red squares) correspond to the characteristic reflections of face-centered cubic (fcc) gold.

Figure 4 shows the XRD pattern of Au@2-propynylamine NPs. The prominent diffraction peaks at 38.2° (111), 44.4° (200), 64.6° (220), and 77.5° (311) correspond to the characteristic reflections of face-centered cubic (fcc) gold, consistent with previous reports [42,43]. Although minor features are visible near 44.7° and 65.0°, the signal-to-noise ratio of the XRD data is insufficient to confidently assign these to impurity phases such as alpha iron (α-Fe). Thus, all observed peaks can be ascribed to fcc gold, and the XRD analysis confirms gold as the predominant crystalline phase in the sample. Comprehensive Structural and Microstructural Analysis of Au@2-propynylamine NPs by XRD, including crystallite size estimation, lattice parameter determination, and Williamson–Hall analysis, is provided in the Supporting Information (Figure S8, Tables S2–S5).

### 3.3. X-Ray Photoelectron Spectroscopy

**Figure 5:**
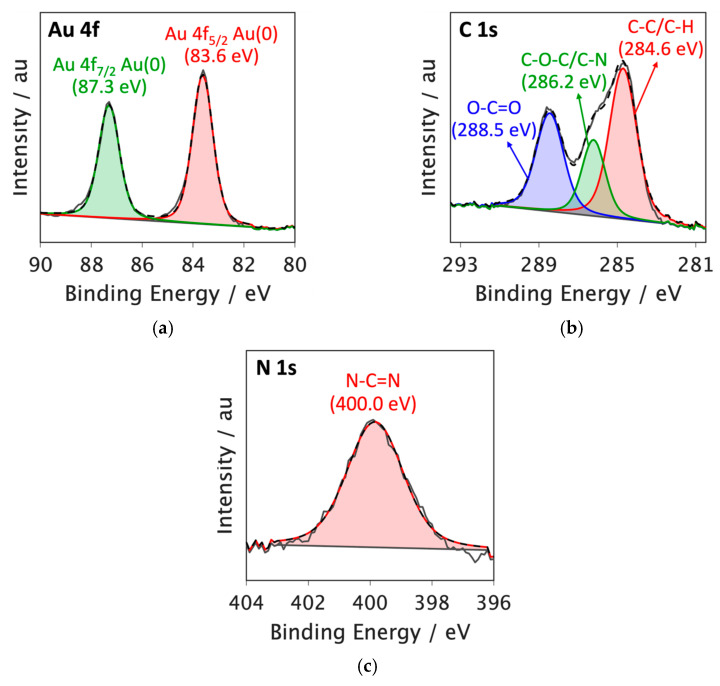
XPS spectra of Au@2-propynylamine NPs produced by RHEBM in water: (**a**) Au 4f (Au 0); (**b**) C1s and (**c**) N1s.

The high-resolution XPS spectrum of the Au 4f core level in the Au@2-propynylamine NPs is displayed in Figure 5a. The spectrum shows two primary peaks attributed to the spin–orbit coupling of Au 4f_7/2_ (83.6 eV) and Au 4f_5/2_ (87.3 eV), which can be attributed to elemental gold in the Au (0) oxidation state [44,45]. Notably, the XPS spectra showed no signs of Au (I) or the gold precursor, Au (III).

As seen in Figure 5b, the C 1s XPS spectrum of Au@2-propynylamine NPs displays deconvoluted peaks at 284.6 eV attributed to C–C/C–H bonds. The peak at 286.2 eV corresponds to C–O–C/C–N bonds, while the deconvoluted peak at 288.5 eV is associated with O–C=O bonds. This component is attributed to adventitious carbon species (e.g., carbonates or esters) adsorbed from the ambient environment during sample preparation and handling, rather than from the ligand or the precursor. The presence of such O–C=O features is commonly reported in XPS studies of air-exposed surfaces [46]. These features, together with the C–O–C peak, confirm the functionalization of the nanoparticles with nitrogen- and oxygen-containing groups from 2-propynylamine. The observed chemical environment also provides evidence for specific interactions between the ligand and the nanoparticle surface, including the coordination of the nitrogen atom from 2-propynylamine to surface gold atoms (Au–N bond), as well as possible hydrogen bonding and dipole interactions involving oxygen-containing groups. These interactions facilitate the anchoring and stabilization of the ligand on the nanoparticle, enhancing both the colloidal stability and the surface reactivity of the Au@2-propynylamine NPs [47].

The N 1s XPS spectrum of Au@2-propynylamine NPs (Figure 5c) exhibits a peak at 400.0 eV, consistent with N–C=N bonds, confirming the interaction of the nitrogen-containing ligand with the nanoparticle surface [48,49].

While no Au (I) or Au (III) species were detected, additional high-resolution XPS analysis (Figure S9, Supporting Information) revealed characteristic peaks for Ni 2p, Fe 2p, Cl 2p, and O 1s, with corresponding assignments summarized in Table S6, Supporting Information. The detected Fe was attributed to contamination from ball milling, Cl to residual gold precursor (HAuCl_4_), and O to oxygen-containing groups from 2-propynylamine and possible surface oxides/hydroxides. These results confirm the predominance of metallic Au (0) while identifying trace impurities and their likely origins.

### 3.4. Ligand Structure at the NP Interface

**Figure 6:**
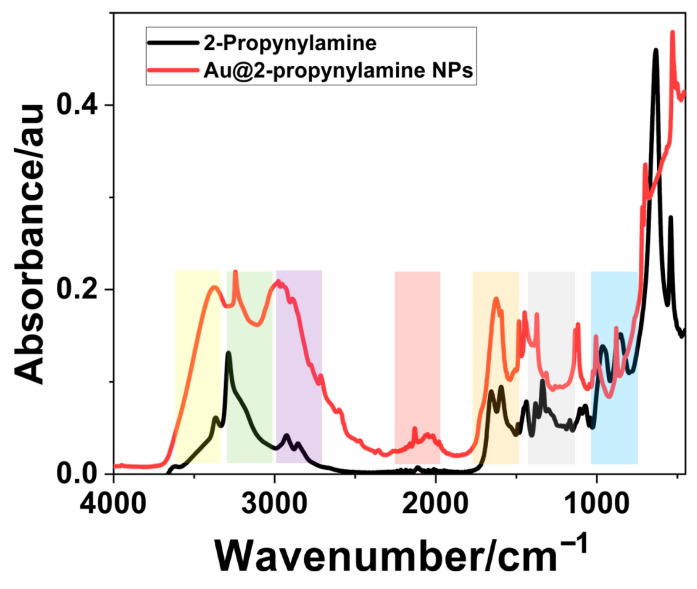
ATR-FTIR spectra of ligand-functionalized nanoparticles synthesized in water (red) compared to free ligand (black). Colored bands are identified in Table 1.

The FTIR-ATR spectra of neat 2-propynylamine (black) and Au@2-propynylamine NPs (red) are shown in Figure 6. The assignments of the observed bands that originate from both 2-propynylamine and Au@2-propynylamine NPs are presented in Table 1. 2-propynylamine bands are assigned based on the literature [50], and nanoparticle bands are assigned based on analogy.

The functionalized AuNPs show a ≡C-H stretching vibration at 3375 cm^−1^, shifted from 3370 cm^−1^ in the free ligand. Similarly, the C≡C stretching appears at 2130 cm^−1^ for the nanoparticle, compared to 2112 cm^−1^ for the free ligand. The broad vibrational bands observed in the nanoparticles are likely due to the surface inhomogeneities. The N-H stretch at 3238 cm^−1^ appears particularly broad in the nanoparticle spectrum, shifted from 3286 cm^−1^ in the free ligand. The region spanning 2850–2950 cm^−1^ corresponds to aliphatic C-H stretching vibrations, while the absorbances at 1670, 880, 600, and 700 cm^−1^ are attributed to low-energy bond deformation and bending modes of the aliphatic amine [51,52].

**Figure 7:**
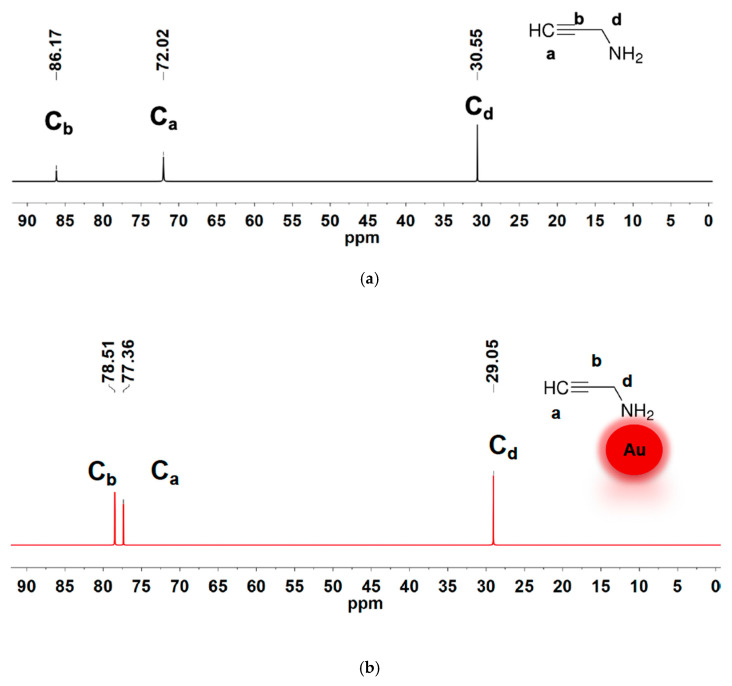
^13^C NMR spectra for: (**a**) 2-propynylamine in DMSO-d_6_ and (**b**) freshly synthesized Au@2-propynylamine NPs in water (shown in red).

**Figure 8:**
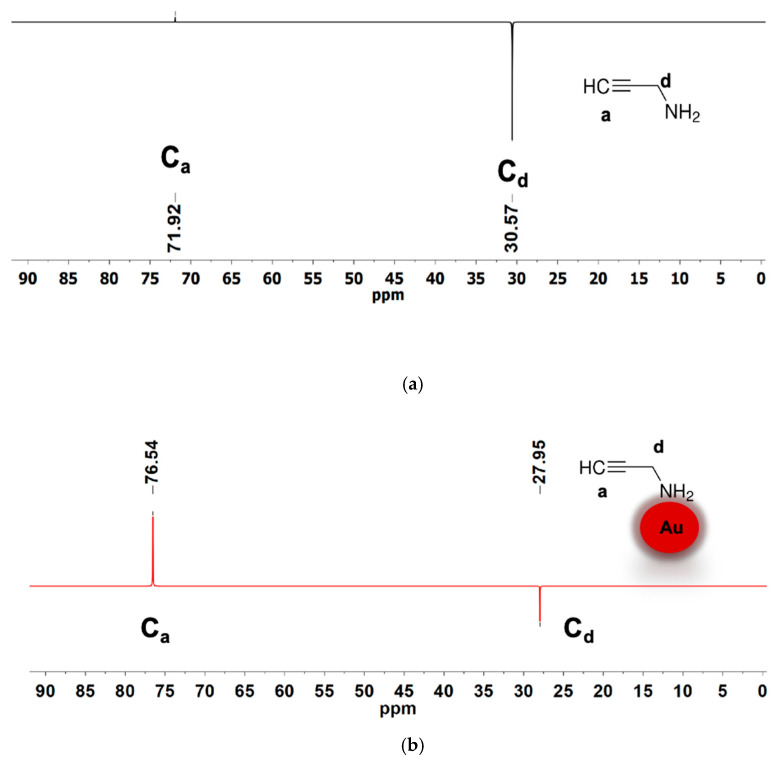
DEPT-135 in DMSO-d_6_ NMR spectra of (**a**) 2-propynylamine and (**b**) Au@2-propynylamine NPs in water.

Figure 7 shows the ^13^C{1H} NMR spectra of the free ligand (Figure 7a) and the ligand coordinated with the gold nanoparticle (Figure 7b). The free ligand spectrum has three ^13^C{1H} resonance signals with chemical shift values of 30.55 ppm, 72.02 ppm, and 86.17 ppm. Upon binding of 2-propynylamine to the AuNP surface, three carbon resonances are observed, with significantly altered chemical shifts. From the DEPT-135 spectrum (Figure 8), the ^13^C{1H} resonances in Figure 7b are assigned as follows: 29.1 ppm (CH_2_), 77.4 (q), 78.5 ppm (CH). The presence of all three carbons with slightly altered chemical shifts relative to the free ligand indicates the ligand remains essentially intact upon coordination. This also suggests that the amine ligand is bound to the gold surface through the nitrogen atom.

Figure 8 presents the DEPT-135 spectra of the free ligand and the nanoparticle. These spectra show clear changes in chemical shifts for both the CH and CH_2_ carbons of 2-propynylamine upon binding to AuNPs. Specifically, the CH peak shifts from 71.92 ppm to 76.54 ppm, and the CH_2_ peak shifts from 30.57 ppm to 27.95 ppm, indicating significant changes in the electronic environment due to the ligand binding on the gold surface. This interaction likely occurs through the nitrogen atom of the amine group, as evidenced by the consistent presence of both carbon resonances in the bound form. The DEPT spectra provide a clear indication of these binding interactions, confirming the successful coordination of 2-propynylamine to the AuNPs.

**Figure 9:**
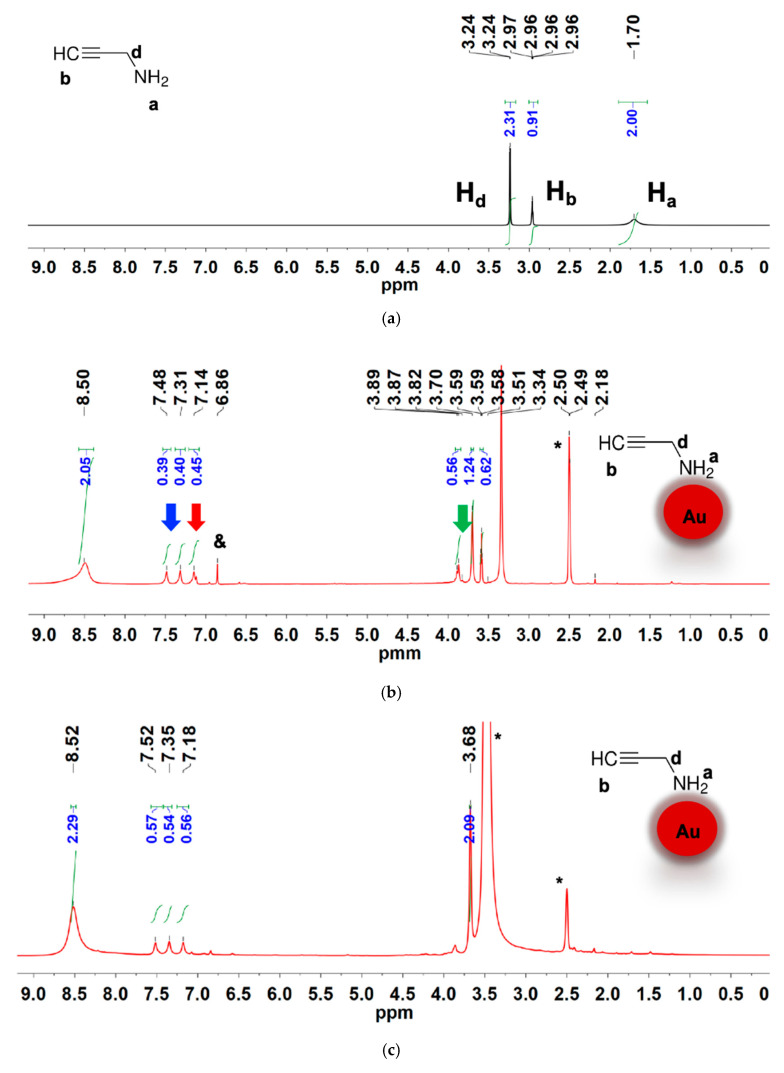
^1^H NMR spectra in DMSO-d_6_ for: (**a**) 2-propynylamine, (**b**) freshly synthesized Au@2-propynylamine NPs, and (**c**) Au@2-propynylamine NPs after six months. The amine proton shifts from 1.70 ppm in the free ligand to 8.50 ppm (blue arrow) upon coordination to AuNPs. Additional signals at 7.48–6.86 ppm (red arrows) indicate minor species, while peaks near 3.89 ppm (green arrow) suggest side products. Asterisks (*) denote residual DMSO-d_6_, and ampersands (&) mark impurities.

Figure 9a–c show the ^1^H NMR spectra of the free 2-propynylamine, the amine ligand coordinated to freshly prepared AuNPs, and the same Au@2-propynylamine NPs after 6 months, respectively. In the free ligand spectrum, the alkynyl CH resonance is observed at 2.96 ppm, while the CH_2_ peak is observed at 3.26 ppm. The much broader amine proton is observed at 1.70 ppm. The spectrum in Figure 9b shows noticeable changes in the chemical shifts when the 2-propynylamine ligand is coordinated to the AuNPs. The most prominent change is the large downfield shift in the amine proton signal from 1.70 ppm in the free ligand to 8.50 ppm, indicated by the blue arrow, which is attributed to a strong metal–ligand interaction with the gold surface [53]. This substantial shift reflects a pronounced change in the electronic environment upon coordination [54]. In addition to the main ligand resonances, new signals appear in the 7.48–6.86 ppm region. These are marked with red arrows in Figure 9b and may correspond to protons from imine-type species or other minor degradation products formed during synthesis. The broad multiplet centered at ~7.31 ppm, also highlighted with a red arrow, exhibits a 1:1:1 splitting pattern characteristic of ammonium ions (^14^NH_4_^+^), suggesting the presence of a small amount of ammonium salt.

Furthermore, small peaks at ~3.89 ppm are marked with a green arrow and may be assigned to possible ligand degradation products or residual side species. The presence of these additional signals supports the proposed mechanism involving partial oxidative transformation of the ligand during nanoparticle formation.

A downfield 1:1:1 triplet is present in the ^1^H NMR spectrum, centered at 7.31 ppm with a spacing of 51 Hz. This pattern is characteristic of ammonium ions (^14^NH^4+^), resulting from spin–spin coupling between the protons and the ^14^N nucleus [55,56,57]. This characteristic splitting pattern arises because the ^14^N nucleus, which has a nuclear spin of 1, can couple with each proton, resulting in three distinct magnetic environments for the protons. These environments lead to the observed triplet in the NMR spectrum, where the intensity ratio of the peaks (1:1:1) reflects the equal probability of the nitrogen spin states [58,59]. The presence of an ammonium ion is also supported by the observation of vibrational modes at 1470–1448 cm^−1^ [58]. The appearance of the ammonium ion in the ^1^H NMR and infrared spectra is likely due to a small amount of ammonium salt, presumably chloride, entrained in our nanoparticles.

The generation of ammonium ions may also occur as a byproduct of the oxidation reaction of 2-propynylamine using gold salt in a ball milling medium during the synthesis of AuNPs. This reaction is a concomitant oxidation process in which a primary amine is converted into an imine through the stoichiometric reduction of Au (III) to Au (0) [60]. This type of reaction is crucial in organic chemistry, as imines are versatile intermediates that can participate in additional transformations, such as the formation of more complex nitrogen-containing compounds or hydrolysis to aldehydes. The reaction can follow different pathways depending on the experimental conditions, such as the presence of water or other amines [61,62].

**Figure 10:**
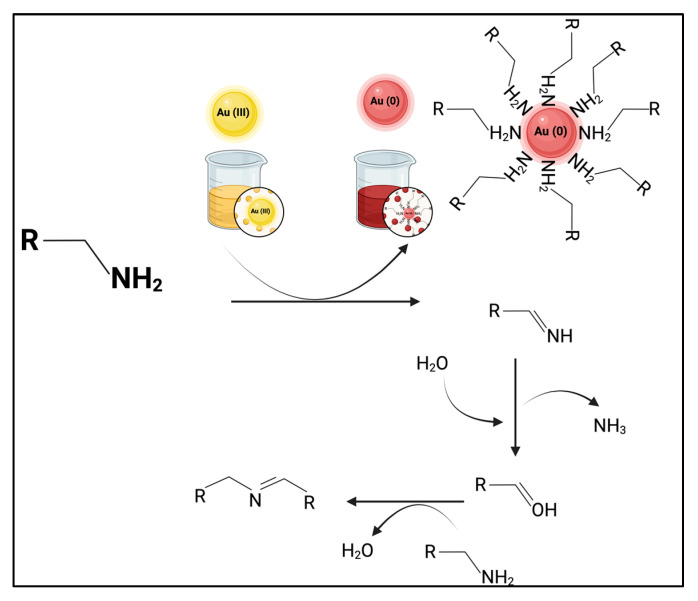
Schematic illustration of proposed mechanism for oxidation of primary amines by gold: formation of imines, AuNPs, and ammonium ions.

Figure 10 illustrates a proposed mechanism for the oxidation of 2-propynylamine by gold salt in a ball-milling medium. In this reaction, 2-propynylamine acts as a reducing agent in the presence of Au (III). The amine loses a hydrogen atom, forming an imine intermediate (HC≡CCH=NH). As the reaction proceeds, Au (III) is reduced to Au (0), resulting in the formation of AuNPs. The hydrolysis of the intermediate imine can also release ammonia (NH_3_) as a byproduct, which is converted to ammonium ions (NH_4_^+^) in the presence of water. In agreement with our ^1^H-NMR observations, these ammonium ions are likely to remain electrostatically associated with the AuNP surface, particularly within the Stern layer of the electrical double layer, where they help stabilize surface charge and colloidal dispersion. As reported in other nanoparticle systems, such surface-bound ions can persist despite repeated aqueous washing steps, and their complete removal generally requires more rigorous purification methods [63,64,65]. The dual role of gold as both an oxidant and nanoparticle precursor underscore the interplay between organic chemistry and nanotechnology [8,66,67].

### 3.5. Surface Click Modification of Alkyne-Functionalized AuNPs Using Biotin-PEG_4_-Azide

The spectroscopic and colorimetric characterization of AuNPs throughout the functionalization process provides a comprehensive validation of the click chemistry strategy and the resulting biofunctionalized nanomaterial. Each spectrum was analyzed in detail to elucidate the physicochemical changes associated with each step.

**Figure 11:**
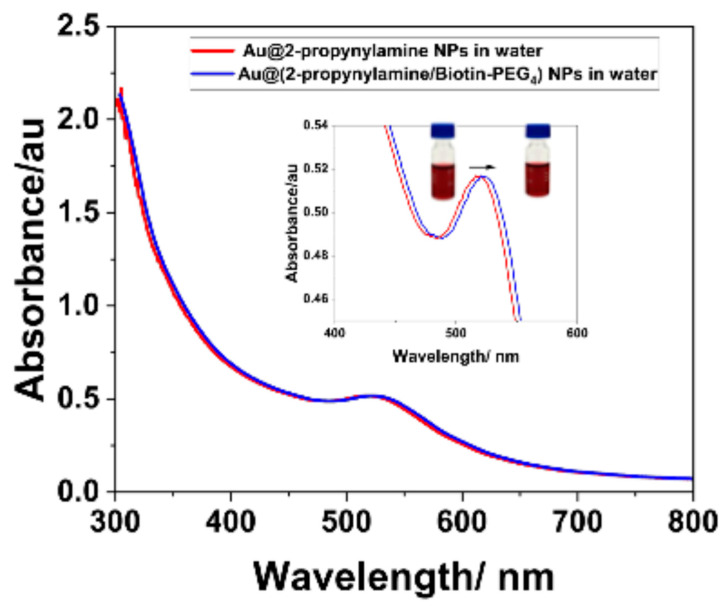
UV-Vis absorbance spectra of AuNPs before and after “click” functionalization.

In Figure 11 (red spectrum), the initial UV–Vis spectrum of Au@2-propynylamine NPs displays a sharp and symmetric surface plasmon resonance (SPR) peak centered at 520 nm, characteristic of a highly monodisperse and optically stable colloidal suspension. Complementary dynamic light scattering (DLS) measurements further support this observation, yielding hydrodynamic size and polydispersity index values indicative of well-dispersed nanoparticles in both DI water and PBS (Table S7 and S8, Supporting Information). The corresponding normalized intensity autocorrelation functions are presented in Figure S10 (Supporting Information), illustrating the faster decay observed for the DI water dispersion—consistent with smaller, well-dispersed nanoparticles—and the slower decay for the PBS dispersion, indicative of larger hydrodynamic size and reduced Brownian diffusion under high ionic strength conditions.

Notably, the localized surface plasmon resonance (LSPR) band remained unchanged in PBS compared to water, further supporting the absence of significant aggregation under physiological ionic strength. This spectral signature confirms the successful synthesis and dispersion of AuNPs functionalized with terminal alkyne groups, establishing a robust foundation for subsequent click chemistry reactions. Upon performing CuAAC with Biotin-PEG_4_-azide, a distinct red shift in the SPR peak to 524 nm is observed (blue spectrum) for the Au@(2-propynylamine/Biotin-PEG_4_) NPs. Notably, the maximum absorbance remains largely unchanged, underscoring the preservation of nanoparticle concentration and colloidal stability throughout the functionalization process.

DLS analysis revealed distinct hydrodynamic behavior: in DI water, nanoparticles showed a narrower size distribution shifted toward smaller diameters, whereas in PBS, distributions were broader and centered at larger sizes, consistent with partial aggregation under high ionic strength (Table S8, Figure S12). These complementary spectroscopic and scattering results confirm that while the LSPR band remains largely unaffected in PBS, subtle aggregation processes can be detected by DLS, highlighting the method’s sensitivity to changes in colloidal stability under physiologically relevant conditions.

**Figure 12:**
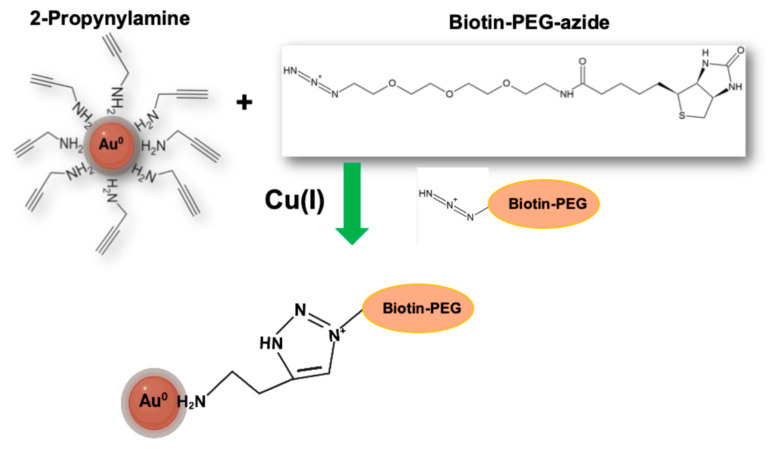
Schematic representation of the functionalization of Au@2-propynylamine NPs with Biotin-PEG_4_-azide via copper(I)-catalyzed azide-alkyne cycloaddition (CuAAC).

This spectral evolution is characteristic of effective surface modification; the covalent attachment of the biotin-PEG_4_ ligand increases the local refractive index at the nanoparticle interface, while the minimal broadening and sustained peak intensity confirm that no significant aggregation or destabilization has occurred [68,69]. The suspension consistently retained its intense burgundy-colored after functionalization, providing further visual evidence of the maintenance of colloidal integrity. Figure 12 schematically illustrates the functionalization process of Au@2-propynylamine NPs with Biotin-PEG_4_-azide via copper(I)-catalyzed azide–alkyne cycloaddition (CuAAC, “click chemistry”). The representation highlights the covalent linkage formed between the terminal alkyne groups on the nanoparticle surface and the azide moiety of the biotin-PEG_4_ ligand, yielding a stable triazole ring. This visual scheme complements the UV–Vis and IR spectroscopic data by providing a molecular-level view of the surface modification process, supporting the successful conjugation demonstrated experimentally.

In control experiments C1, C2, and C3, the UV-Vis spectra showed no changes in the SPR band of the AuNPs; the SPR peak remained sharp and centered around 520 nm, indicating that the nanoparticles retained their optical properties and colloidal stability under each condition. Control C4 did not display a SPR band because this condition did not contain AuNPs. This result confirms that no optical changes or aggregation occurred in the controls with nanoparticles, and that the absence of the SPR signal in C4 was solely due to the lack of nanoparticles in the system.

**Figure 13:**
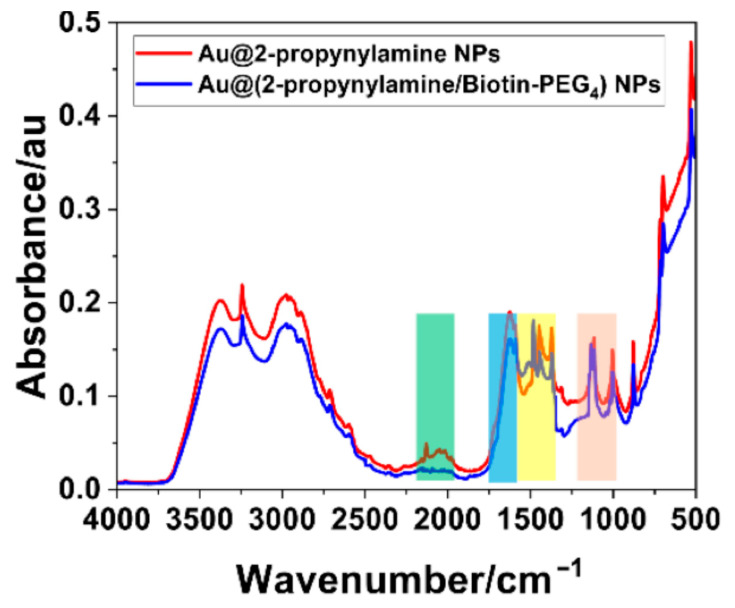
FTIR spectra of Au@2-propynylamine NPs before (red) and after (blue) click functionalization with Biotin-PEG_4_-azide. The disappearance of the ~2100 cm^−1^ band, highlighted in the green-shaded region (2000–2200 cm^−1^), confirms the consumption of terminal alkynes. Additional vibrational changes are observed in the light-blue region (1650–1680 cm^−1^, amide C=O of biotin), the yellow region (1350–1450 cm^−1^, C–N stretching and CH_2_ wagging from triazole/PEG), and the beige region (1100–1250 cm^−1^, C–O–C stretching of the PEG_4_ linker).

The chemical transformation of AuNPs functionalized with terminal alkyne groups was confirmed by FTIR spectroscopy, performed before and after the CuAAC reaction with Biotin-PEG_4_-azide, as illustrated in Figure 13. The spectrum in red, corresponding to AuNPs passivated with 2-propynylamine, displays a prominent absorption band centered around 2100 cm^−1^. This feature is characteristic of the C≡C stretching vibration of terminal alkynes and serves as a distinct spectroscopic marker for monitoring the presence and reactivity of alkynyl groups on the nanoparticle surface [51,52]. The intensity and sharpness of this peak further confirm the accessibility of reactive alkyne functionalities, which are essential for subsequent click chemistry.

The blue spectrum in Figure 13 shows the disappearance of the ~2100 cm^−1^ band following the CuAAC reaction with Biotin-PEG_4_-azide, confirming the consumption of terminal alkynes [70]. This spectral change is consistent with the formation of 1,2,3-triazole linkages, demonstrating that the click reaction proceeded efficiently and selectively on the AuNP surface. Consistently, the green-shaded region (2000–2200 cm^−1^) shows a marked reduction in intensity, further supporting the loss of terminal alkynes. Additional vibrational changes are highlighted in Figure 13: the light-blue region (1650–1680 cm^−1^), corresponding to amide C=O stretching from the biotin moiety [71,72]; the yellow region (1350–1450 cm^−1^), associated with C–N stretching and CH_2_ wagging from the triazole ring and PEG spacer; and the beige region (1100–1250 cm^−1^), assigned to C–O–C stretching of the PEG_4_ linker [73,74]. Although these features are subtle due to the monolayer nature of the coating and overlapping vibrational modes, their presence is consistent with literature reports of PEG/biotin-functionalized noble-metal nanoparticles. A complete vibrational band assignment is provided in Table S9 (Supporting Information).

**Figure 14:**
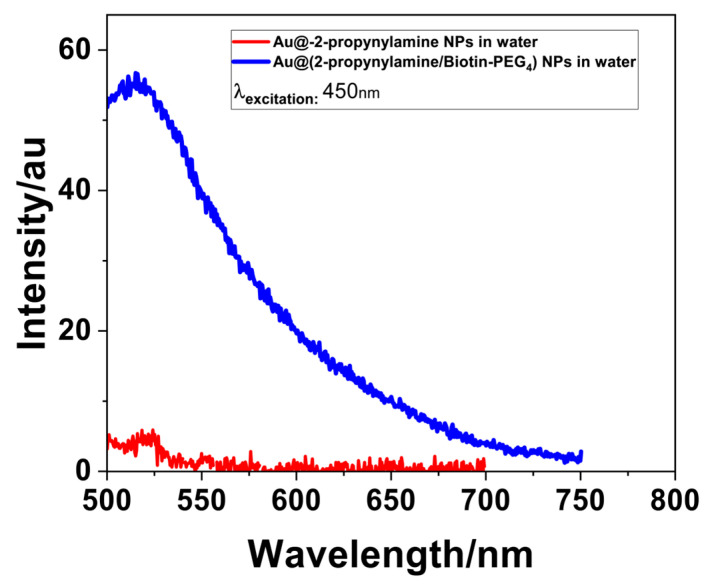
Photoluminescence spectra of Au@2-propynylamine NPs before and after click functionalization with Biotin-PEG_4_-azide (λ_exc_ = 450 nm).

Photoluminescence spectroscopy was employed to evaluate the optical consequences of surface modification on the AuNPs, using an excitation wavelength of 450 nm. As shown in Figure 14, the unmodified Au@2-propynylamine NPs—stabilized with 2-propynylamine ligands and presenting terminal alkyne groups—exhibited negligible emission across the visible spectrum. This observation is consistent with the well-established quenching behavior of bare and alkynyl-passivated gold surfaces, dominated by non-radiative decay pathways such as plasmonic dephasing and surface trap recombination [75,76,77]. In contrast, following copper-catalyzed azide–alkyne cycloaddition (CuAAC) with Biotin-PEG_4_-azide, the resulting Au@2-propynylamine NPs /Biotin-PEG_4_ conjugates displayed a pronounced and broad fluorescence emission extending from approximately 500 to 750 nm, with maximum intensity centered around 525–530 nm and a more than tenfold increase in signal intensity.

This enhancement is attributed to the synergistic effects of surface passivation by the Biotin-PEG_4_ corona, which reduces non-radiative energy loss by minimizing trap states at the nanoparticle interface [68,78], as well as modification of the local dielectric environment, which influences radiative decay rates by altering the refractive index landscape around the gold core [79]. The absence of sharp vibronic features and the emission’s strong dependence on surface chemistry initially suggested that the photoluminescence could arise from hybridized surface states at the nanoparticle interface or from emissive triazole moieties formed via the click reaction [80,81].

Control photoluminescence experiments were conducted on each individual component to determine the origin of the observed emission. Neither free 2-propynylamine nor Biotin-PEG_4_-azide exhibited any detectable fluorescence under the same experimental conditions. This finding is in full agreement with theoretical and literature reports, which consistently demonstrate that both 2-propynylamine and biotin–PEG derivatives lack intrinsic fluorescence and require conjugation with an external fluorophore for detection [82,83]. In addition, a control CuAAC reaction between Biotin-PEG_4_-azide and propargylamine in the absence of AuNPs yielded a non-fluorescent triazole product [84]. Collectively, these results confirm that the photoluminescence observed in the Au@2-propynylamine NPs/Biotin-PEG_4_ system originates from newly formed hybridized surface states at the gold–triazole interface, rather than from any individual reagent or the free triazole in solution. To minimize contributions from unbound species, all nanoparticle samples were thoroughly washed and centrifuged following functionalization [84,85,86,87,88].

### 3.6. Colorimetric Assay (NP-ELISA)

**Figure 15:**
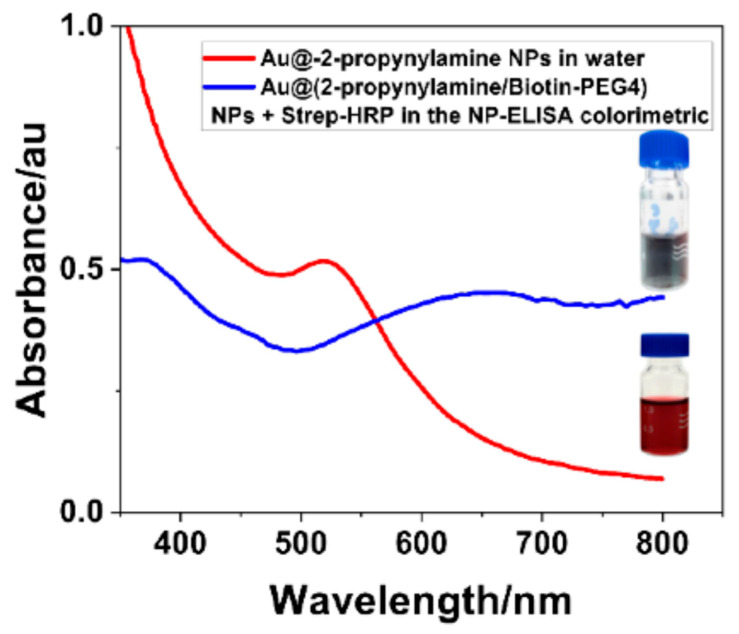
UV-Vis spectra of functionalized AuNPs before and after HRP–biotin–streptavidin coupling. The blue spectrum represents Au@(2-propynylamine/Biotin-PEG4) NPs after interaction with streptavidin-HRP in the NP-ELISA assay, resulting in a shifted, broadened spectrum and a visible color change to dark gray/blue (top vial). The red spectrum corresponds to Au@2-propynylamine NPs in water, showing the characteristic plasmon peak near 520 nm and a burgundy-colored solution (bottom vial).

The UV-Vis spectra shown in Figure 15 offer clear optical evidence of successful AuNP surface functionalization with Biotin-PEG_4_, as well as the specific biorecognition events driving enzymatic signal generation in the NP-ELISA platform [27]. The red trace, representing unmodified Au@2-propynylamine NPs, shows the characteristic SPR band expected for stable colloidal gold, confirming their unfunctionalized state. By contrast, the blue trace—corresponding to Au@(2-propynylamine/Biotin-PEG_4_) NPs incubated with streptavidin–HRP and the chromogenic substrate TMB—displays two distinct features that signify enzymatic activity: (1) a shoulder at ~370–380 nm linked to the formation of the TMB•^+^ radical cation [27,30,68], and (2) a broad, intense band around 650–700 nm, associated with the fully oxidized diimine product (TMBox) responsible for the deep blue coloration [89]. This spectral fingerprint aligns with visual evidence; the vial on the left in Figure 15 (functionalized AuNPs + HRP–TMB) shows a strong blue/black coloration, indicative of high TMB turnover and effective biotin–streptavidin interaction [27,90]. The vial on the right, containing non-functionalized AuNPs under the same assay conditions, retains a burgundy color. This confirmed the lack of HRP binding and enzymatic reaction. This contrast provides an intuitive, real-time readout of surface functionalization and assay specificity.

Overall, the evolution of each spectrum and colorimetric response provides robust, multi-modal evidence for the efficient, selective, and stable click functionalization of AuNPs. The results demonstrate that the protocol described in Section 2.2.6 yields biotinylated AuNPs with preserved colloidal stability and fully accessible surface ligands, suitable for biosensing and advanced bioanalytical applications. Zeta potential measurements were not performed in this work, as the study focused on spectroscopic and optical characterization. Future larger-scale preparations will incorporate surface charge analysis to complement the current multi-technique characterization.

## 4. Conclusions

This work introduces a rapid, scalable, and environmentally benign strategy for synthesizing alkyne-functionalized AuNPs via RHEBM. By harnessing 2-propynylamine in the dual roles of reducing agent and surface ligand, we achieved aqueous preparation of monodisperse, click-ready AuNPs (~4 nm) within 15 min. The resulting nanoparticles display terminal alkyne functionalities (–C≡CH) that are readily accessible for subsequent surface modifications, enabling efficient bioconjugation through CuAAC.

The synthesized AuNPs exhibit remarkable colloidal stability when subjected to variations in temperature and pH, conditions that are relevant for potential biological and diagnostic applications. This robustness underscores their suitability for further integration into biosensing and delivery platforms. The success of post-synthetic click functionalization was thoroughly confirmed through a combination of spectroscopic, photoluminescent, and colorimetric analyses, which collectively demonstrated not only the chemical reactivity of the engineered nanomaterials but also their sustained dispersibility and lack of aggregation under simulated physiological environments. Although this study does not include direct in vivo or cell-based testing, the observed nanoparticle stability and functionalization efficiency under temperature and pH variations are strong indicators of their compatibility with future biological applications.

This mechanochemical approach significantly streamlines AuNP synthesis, removing the need for toxic reagents, organic solvents, and laborious multi-step protocols. The seamless integration of mechanochemistry and click chemistry establishes a versatile and sustainable platform for the rapid production and functionalization of AuNPs. This methodology paves the way for the broader adoption of functional nanomaterials in nanoscience, biomedicine, and advanced materials engineering, offering a promising route toward high-throughput and environmentally responsible nanomanufacturing.

## Figures and Tables

**Table 1:** ATR-FTIR vibrational bands and functional groups of Au@2-propynylamine NPs.

2-propynylamine (cm^−1^)	Au@2-propynylamine NPs (cm^−1^)	Assignment
3370 (m)	3375 (br)	≡C-H stretch
3286 (m)	3238 (m)	N-H stretch
2853 (m)	–	C-H stretch
2933 (m)	2956 (m)	C-H stretch
2112 (m)	2130 (m)	C≡C stretch
1670 (m)	1622 (m)	N–H bend
–	1470 and 1448 (w)	(primary amines)
		NH_4_^+^, bending vibrations.
1381 (m)	1373 (m)	C-H, Alkane
1330 (w)	–	C-H, Alkane
1104 (m)	–	
967 (m) 877 (m) 636 (s)	1117 (m) 1006 (m) 880 (m) 700 (s)	C-N, aliphatic amines
		C-H, bending.
		N–H wag
		C-H deformation
		–C≡C–H: C–H
541 (s)	530 (s)	Hydrocarbons C-H and C-C stretching and bending vibrations

Note: s = strong; m = medium; w = weak; br = broad. Colored text corresponds to bands within Figure 6.

## Data Availability

The original contributions presented in the study are included in the article, further inquiries can be directed to the corresponding author.

## Supplementary Materials

The following supporting information can be downloaded at: https://www.mdpi.com/article/10.3390/ma18194470/s1, Figure S1: 1H and 13C NMR spectra of the supernatant; Figure S2: UV–Vis spectrum of the supernatant; Figure S3: FTIR spectrum of the supernatant; Figure S4: synthesis of Au@2-propynylamine NPs at different reaction times; Figure S5: UV–Vis spectra and TEM images of Au@2-propynylamine NPs at different reaction times; Figure S6: UV–Vis spectrum and TEM of Au@2-propynylamine NPs synthesized by RHEBM in zirconia vials; Figure S7: UV–Vis spectrum and TEM of Au@2-propynylamine NPs synthesized by reflux; Figure S8: Williamson–Hall plot for Au@2-propynylamine NPs; Figure S9: XPS spectra of Ni, Fe, O, and Cl for Au@2-propynylamine NPs; Figure S10: DLS autocorrelation functions in PBS and DI water; Figure S11: DLS-derived distributions for Au@2-propynylamine NPs in DI water; Figure S12: DLS-derived distributions for Au@2-propynylamine NPs in PBS; Table S1: Yields of Au@2-propynylamine NPs at different Au:ligand molar ratios; Table S2: XRD peak identification and corrected widths; Table S3: crystallite size from the Scherrer equation; Table S4: lattice parameter calculations; Table S5: microstrain and crystallite size from Williamson–Hall; Table S6: XPS-identified elements, binding energies, and probable origins; Table S7: DLS-derived diameters and distributions in DI water; Table S8: DLS-derived diameters and distributions in PBS; Table S9: FTIR band assignments before and after Biotin-PEG_4_ functionalization.

## Abbreviations

The following abbreviations are used in this manuscript:

AuNPs	Gold nanoparticles
RHEBM	Reactive high-energy ball milling
PBS	Phosphate-buffered saline
SPR	Surface plasmon resonance
CuAAC	Copper(I)-catalyzed azide-alkyne cycloaddition
NP-ELISA	Nanoparticle-based enzyme-linked immunosorbent assay
TMB	3,3′,5,5′-Tetramethylbenzidine
