# Supplementary material for: Click-Ready Gold Nanoparticles from Aqueous Mechanochemistry: 2-Propynylamine as a Reducing Agent and Surface Ligand
Source: Materials (Basel). 2025 Sep 25;18(19):4470. doi: 10.3390/ma18194470 (PMC12526151; doi:10.3390/ma18194470)
Supplement: Supplementary file 1 [file materials-18-04470-s001.zip › materials-3797205-supplementary.pdf]

Supplementary Materials for

Click-Ready Gold Nanoparticles from Aqueous Mechanochemistry: 2-Propynylamine as a Reducing Agent and Surface Ligand

Amber L. Garcia [1], Brian S. Mitchell [2], Amanda Reusch [2], Mark J. Fink [3], Juan P. Hinestroza [4], Yelin Ko [4] and Julie P. Vanegas [5,*]

[1] College of Engineering and Computer Science, The University of Texas Rio Grande Valley, Edinburg, TX 78539, USA; amber.garcia05@utrgv.edu
[2] Department of Chemical and Biomolecular Engineering, Tulane University, New Orleans, LA 70118, USA; brian@tulane.edu (B.S.M.); areusch@tulane.edu (A.R.)
[3] Department of Chemistry, Tulane University, New Orleans, LA 70118, USA; fink@tulane.edu
[4] Department of Human Centered Design, College of Human Ecology, Cornell University, Ithaca, NY 14850, USA; jh433@cornell.edu (J.P.H.); yk563@cornell.edu (Y.K.)
[5] Department of Physics and Astronomy, The University of Texas Rio Grande Valley, Edinburg, TX 78539, USA
* Correspondence: julie.vanegas@utrgv.edu; Tel.: +1-(956)-665-2056

Table S1. Yields of Au@2-propynylamine nanoparticles for different Au:ligand molar ratios.

Au : 2-propynylamine ratio	Replicate 1 (%)	Replicate 2 (%)	Replicate 3 (%)	Mean ± SD (%)
1 : 10	45	60	75	60.0 ± 15.0
1 : 5	32	65	67	54.7 ± 19.6
1 : 3	80	92	91	87.7 ± 6.6

These results demonstrate that the 1:3 ratio offers the best balance between nanoparticle formation, purification efficiency, and reproducibility.

Comprehensive Characterization of the Supernatant by ^{1}H and ^{13}C NMR, UV–Vis Spectroscopy, and FTIR

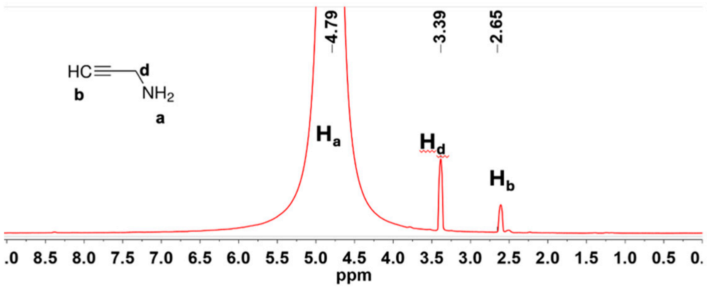

A

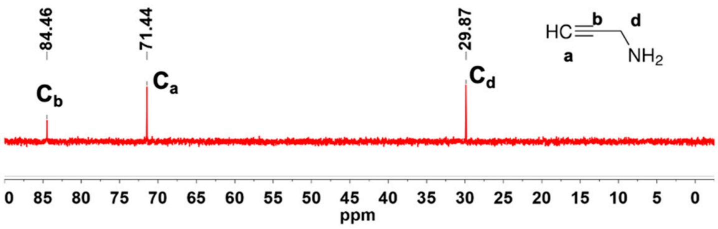

B

Figure S1. (A) ^{1}H and B)^{13}C NMR in DMSO-d6 spectra of the supernatant showing only excess ligand in water after AuNPs removal.

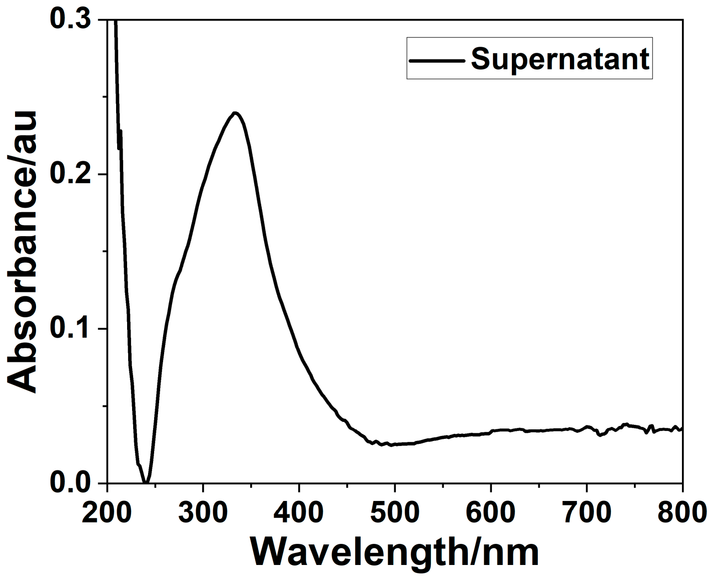

Figure S2. UV–Vis spectroscopy of the supernatant showing absence of AuNPs plasmon band near 520 nm.

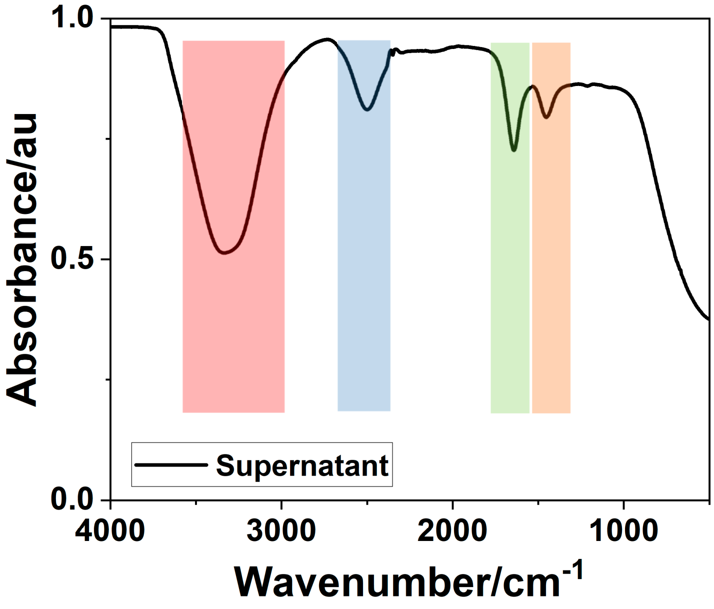

Figure S3. FTIR spectrum of the supernatant after AuNPs removal, showing signals attributed to excess 2-propynylamine in water.

Red zone: O–H stretch from water, which can overlap with or broaden the N–H stretches of the primary amine and the sp-hybridized C–H stretch (~3300 cm^{-1}).

Blue zone: ~2500 cm^{-1}, typically associated with N–H stretching overtone/combination in primary amines when hydrogen-bonded; in aqueous samples, this can also be influenced by O–H···N hydrogen bonding or residual CO_2 in the ATR setup (weak absorption near 2400 cm^{-1}).

Green zone: 1580–1650 cm^{-1}, H–O–H bending mode of water overlapping with the N–H bending of the amine.

Orange zone: 1400–1300 cm^{-1}, likely due to C–H bending vibrations from the ligand.

Time control experiment

RHEBM synthesis conditions were varied with reaction durations of 15, 30, 45, 60, 90, and 120 minutes. 200 mg of chloroauric acid trihydrate and 89.9 mg of 2-propynylamine (1:3 molar ratio) were placed in a hardened stainless steel milling vial with two 5 mm stainless steel balls (0.21 g each) and 30 ml of purified water. The reduction process was driven exclusively by the milling action, with no additional reducing agents.

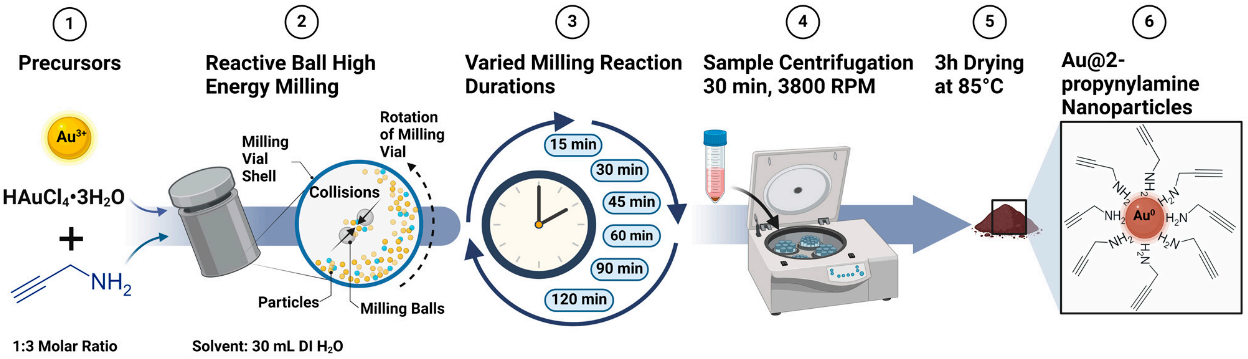

Figure S4. Synthesis of Au@2-propynylamine NPs in different timeframes.

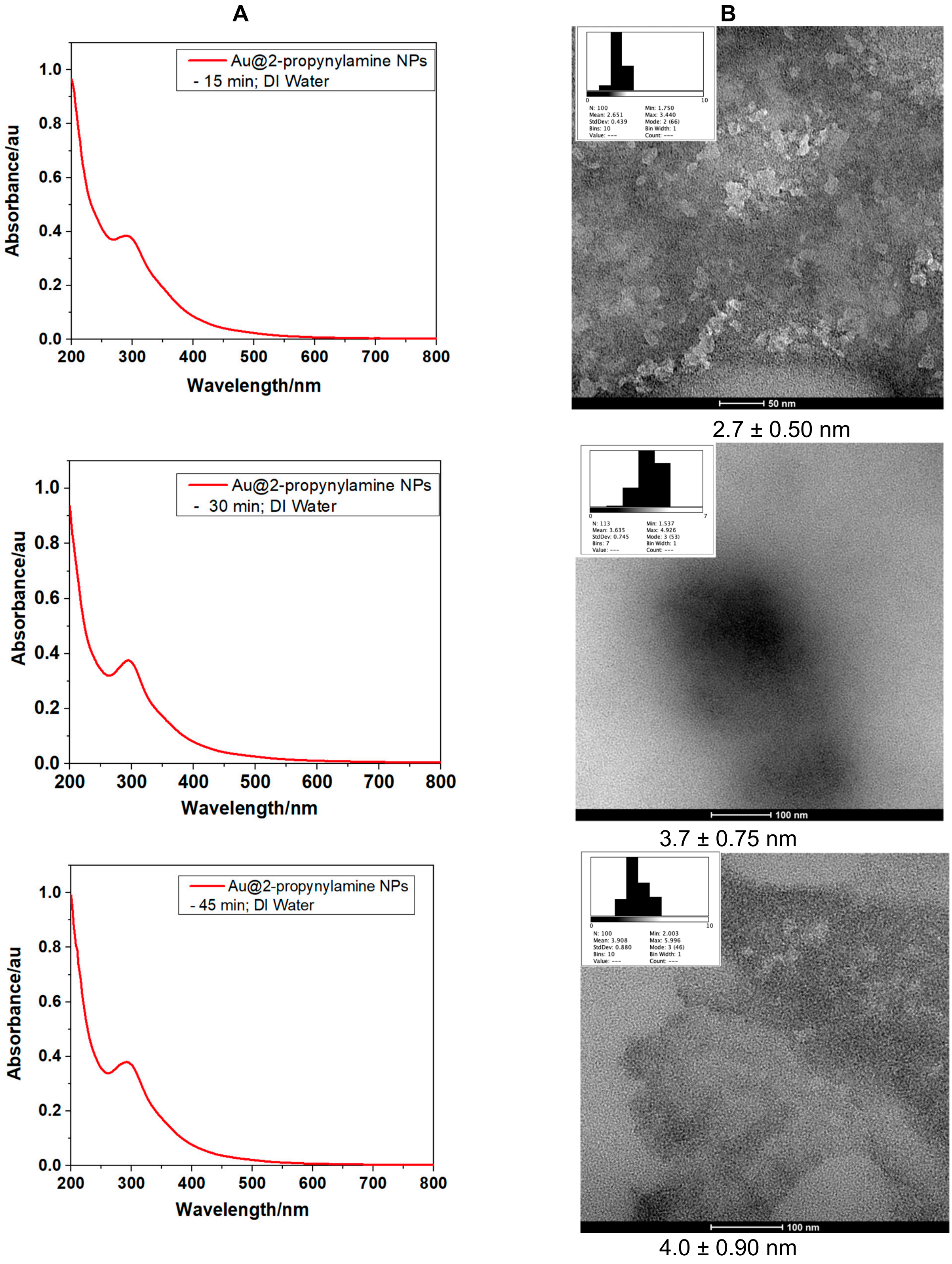
A
B
Au@2-propynylamine NPs
- 15 min; DI Water
Absorbance/au
Wavelength/nm
Au@2-propynylamine NPs
- 30 min; DI Water
Absorbance/au
Wavelength/nm
Au@2-propynylamine NPs
- 45 min; DI Water
Absorbance/au
Wavelength/nm
50 nm
2.7 ± 0.50 nm
100 nm
3.7 ± 0.75 nm
100 nm
4.0 ± 0.90 nm

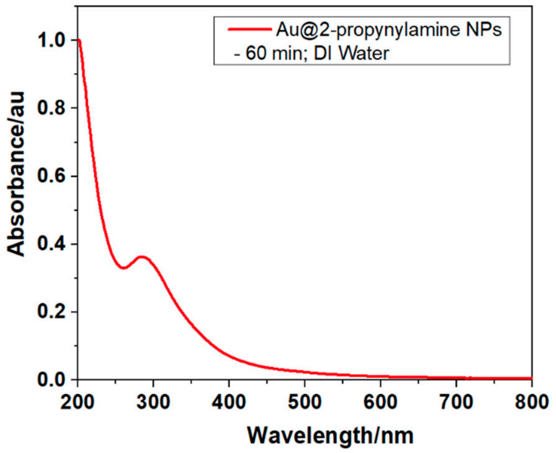

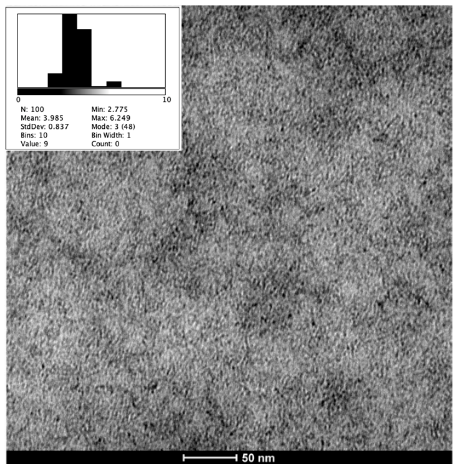

4.0 ± 0.84 nm

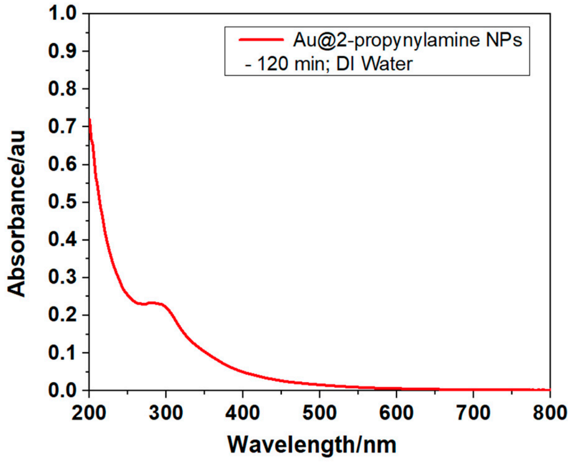

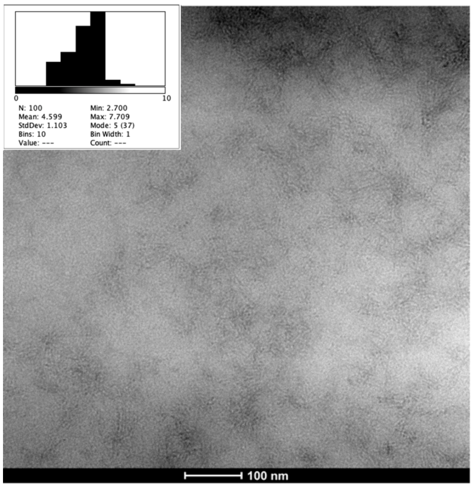

4.6 ± 1.1 nm

Figure S5. A) The UV-visible absorption spectrum of Au@2-propynylamine NPs in the water reaction durations of 15, 30, 45, 60 and 120 minutes, shows the absorption band at 300 nm and B) TEM image of Au@2-propynylamine NPs scale bar of 50 nm in water reaction durations of 15, 30, 45, 60, 90, and 120 minutes.

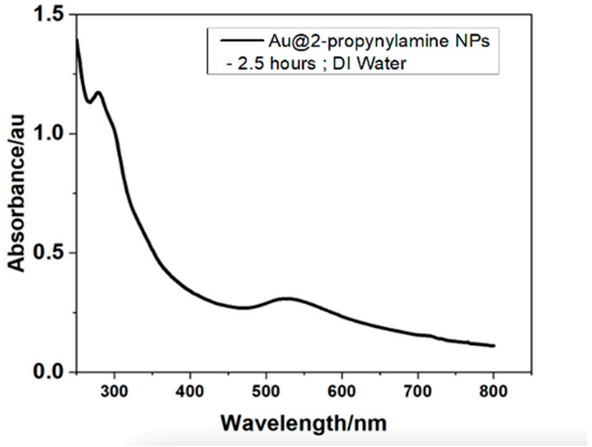

A

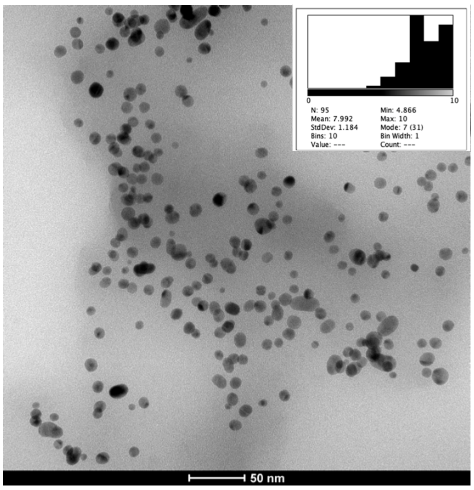

B

8.0 ± 1.2 nm

Figure S6. Au@2-propynylamine nanoparticles in water synthesized by RHEBM in zirconia milling vials for 2.5 hours in a cold room: A) UV-visible absorption spectrum in water. B) TEM image (scale bar :50 nm).

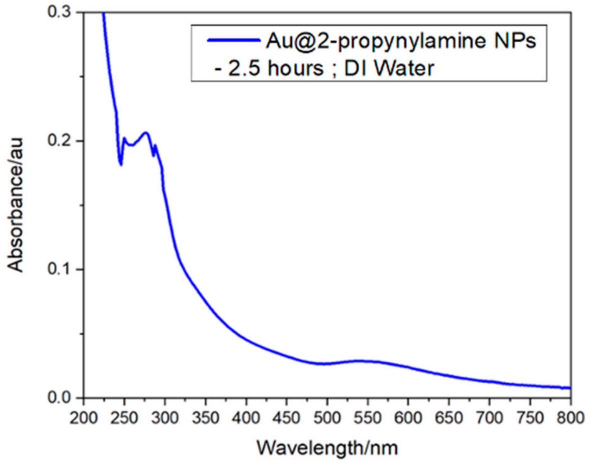

A

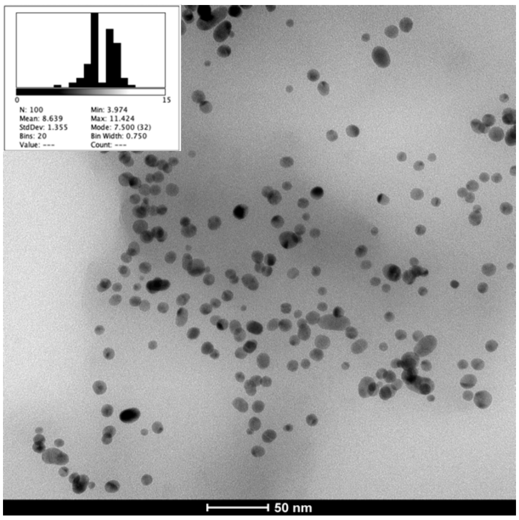

B

9.0 ± 0.5 nm

Figure S7. Au@2-propynylamine nanoparticles in water synthesized by reflux for 3 hours: A) UV-visible absorption spectrum in water. B) TEM image with a scale bar of 50 nm.

Comprehensive Structural and Microstructural Analysis of Au@2-propynylamine NPs by XRD

Table S2 – Peak identification and corrected widths.

hkl	***2θ*** **obs (°)**	**FWHM measured (°)**	**FWHM corrected (°)**	**Maximum Intensity (cps)**
(111)	38.111	0.494	0.484	2115.1
(200)	44.257	0.681	0.673	1448.3
(220)	64.588	0.545	0.536	1267.0
(311)	77.567	0.990	0.985	1208.5

$$D = \frac{K\lambda}{\beta \cos\theta}$$

Where

- D = crystallite size (in Å, converted to nm for the table)
- K = shape factor (we used K=0.9)
- λ = X-ray wavelength (for Cu Kα, λ=1.5406 Å)
- β = corrected full width at half maximum (FWHM) of the peak in radians
- θ = Bragg angle (half of the measured 2θ)

Correction for instrumental broadening: Before using β, the measured FWHM (β_{meas}) was corrected using the following relation: β_{corr} = √ (β_{meas^2} – β_{inst^2}), where β_{inst} was assumed as 0.10° (converted to radians before use).

$$\beta = \sqrt{\beta_{meas}{}^{2} + \beta_{inst}{}^{2}}$$

Where

- β_{inst} = instrumental broadening in degrees (assumed 0.10°), converted to radians before use.

Table S3 – Crystallite size from the Scherrer equation.

hkl	***2θ*** **obs (°)**	**β_{corr} (rad)**	**Crystallite size, D (nm)**
(111)	38.111	0.008447	17.37
(200)	44.257	0.011746	12.74
(220)	64.588	0.009355	17.53
(311)	77.567	0.017191	10.35

Bragg's law to calculate the interplanar spacing (d):

$$d = \frac{\lambda}{2 \sin \theta}$$

Cubic crystal relation to calculate the lattice parameter (a)

$$\mathrm{a} = d\sqrt{l^2 + h^2 + k^2}$$

Where

- λ = X-ray wavelength for Cu Kα radiation (1.5406 Å)
- θ = Bragg angle (half of the measured 2θ
- h, k, l = Miller indices of the reflection
- d = interplanar spacing in Å
- a = lattice parameter in Å

Table S4 – Lattice parameter (a) from each reflection, the calculation was based on Bragg's law and the cubic crystal geometry.

hkl	***d*-spacing (Å)**	**Lattice parameter, a (Å)**
(111)	2.3594	4.0866
(200)	2.0449	4.0899
(220)	1.4418	4.0780
(311)	1.2298	4.0787
**Mean ± SD**		**4.0833 ± 0.0059**

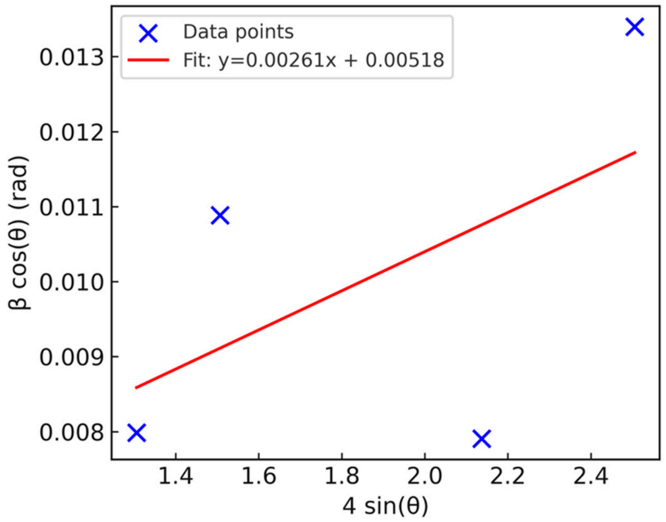

Figure S8. Williamson–Hall plot of β cos θ versus 4 sin θ for Au@2-propynylamine nanoparticles. The slope corresponds to the microstrain (ε) and the intercept to the crystallite size contribution according to the Williamson–Hall equation.

Table S5 – Microstrain and crystallite size from Williamson–Hall.

Microstrain, ε (%).	Crystallite size D_{W-H} (nm)	R^2
0.262	26.83	0.3049

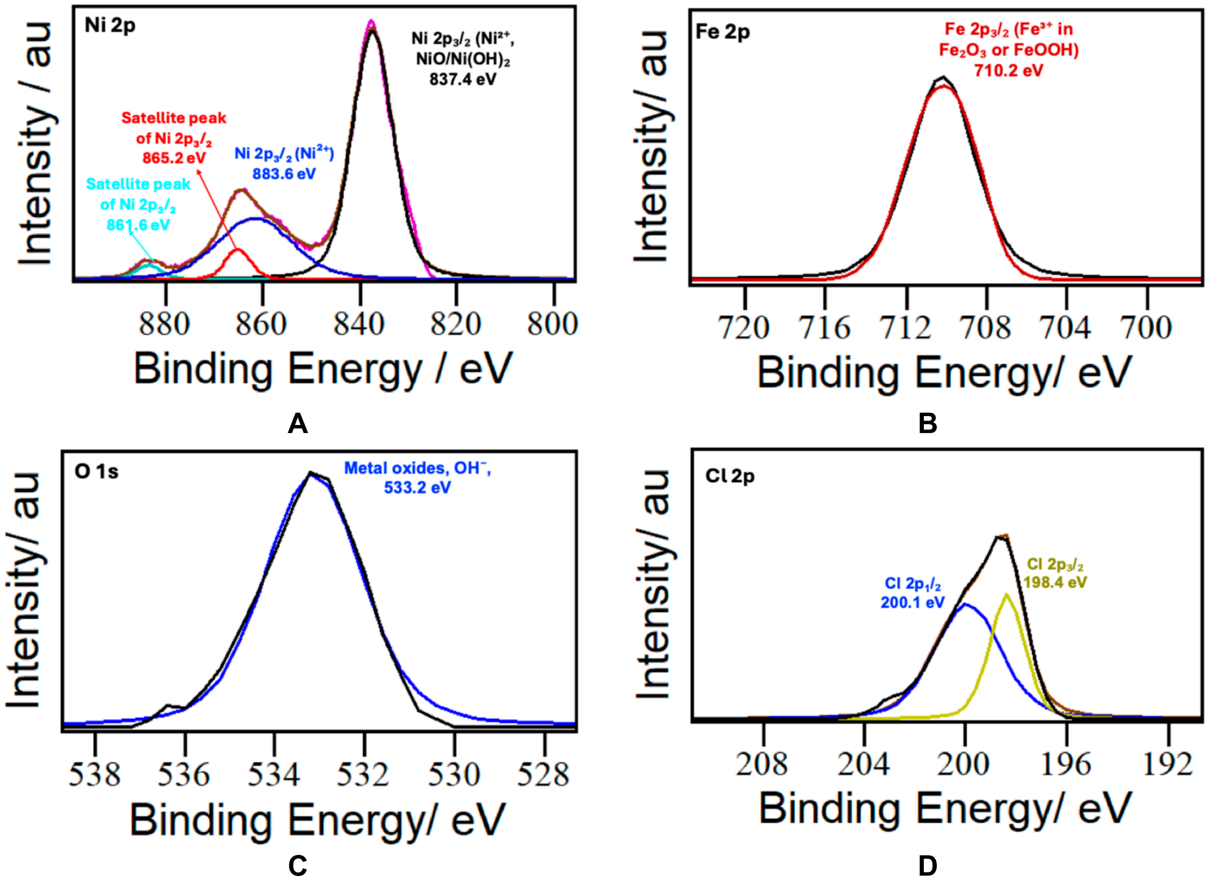

Figure S9. XPS spectra of Au@2-propynylamine nanoparticles: (A) Ni 2p, (B) Fe 2p, (C) O 1s, and (D) Cl 2p.

Table S6. XPS-Identified Elements, Binding Energies, and Probable Origins in Au@2-Propynylamine NP Samples.

Element	Orbital / Main peak	Typical Binding Energy (eV)	Chemical State	Probable Origin
**Ni**	Ni $2p_{3/2}$	852.6–855.0	Ni^{0}, Ni^{2+} (NiO, $Ni(OH)_2$)	Contamination from ball milling (components/alloys containing Ni)
	Ni $2p_{1/2}$	869.0–872.0	Ni^{0}, Ni^{2+}	Contamination from ball milling
**Cu**	Cu $2p_{3/2}$	932.3–934.5	Cu^{0} / Cu^{+}, Cu^{2+} (CuO, $Cu(OH)_2$)	Contamination from adhesive tape used for XPS mounting
	Cu $2p_{1/2}$	952.3–954.5	Cu^{0} / Cu^{+}, Cu^{2+}	Contamination from adhesive tape
**Fe**	Fe $2p_{3/2}$	706.5–711.0	Fe^{0}, Fe^{2+}, Fe^{3+}	Contamination from ball milling (stainless steel)
	Fe $2p_{1/2}$	720.0–724.0	Fe^{0}, Fe^{2+}, Fe^{3+}	Contamination from ball milling
**O**	O 1s	529.5–533.5	Metal oxides, OH^{-}, adsorbed H_2O	Oxygen-containing groups from 2-propynylamine and possible surface oxides/hydroxides
**Cl**	Cl $2p_{3/2}$	198.0–199.5	Cl^{-} (NaCl, adsorbed HCl)	Residue from gold precursor ($HAuCl_4$)
	Cl $2p_{1/2}$	200.0–201.5	Cl^{-}	Residue from gold precursor

Dynamic Light Scattering (DLS) Analysis of Au@2-Propynylamine NPs in water and PBS

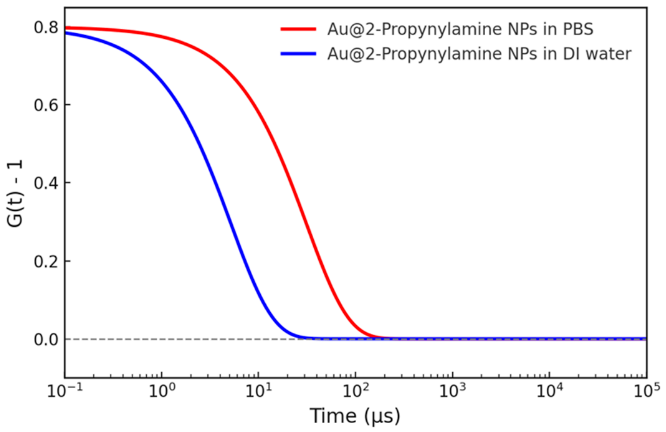

Figure S10. Normalized intensity autocorrelation functions obtained by dynamic light scattering (DLS) for Au@2-propynylamine nanoparticles dispersed in phosphate-buffered saline (PBS, red) and in deionized (DI) water (blue). The PBS dispersion exhibits a slower decay, indicating larger hydrodynamic size and slower Brownian diffusion, consistent with nanoparticle aggregation in high ionic strength medium. In contrast, the DI water dispersion decays more rapidly, consistent with smaller, well-dispersed nanoparticles.

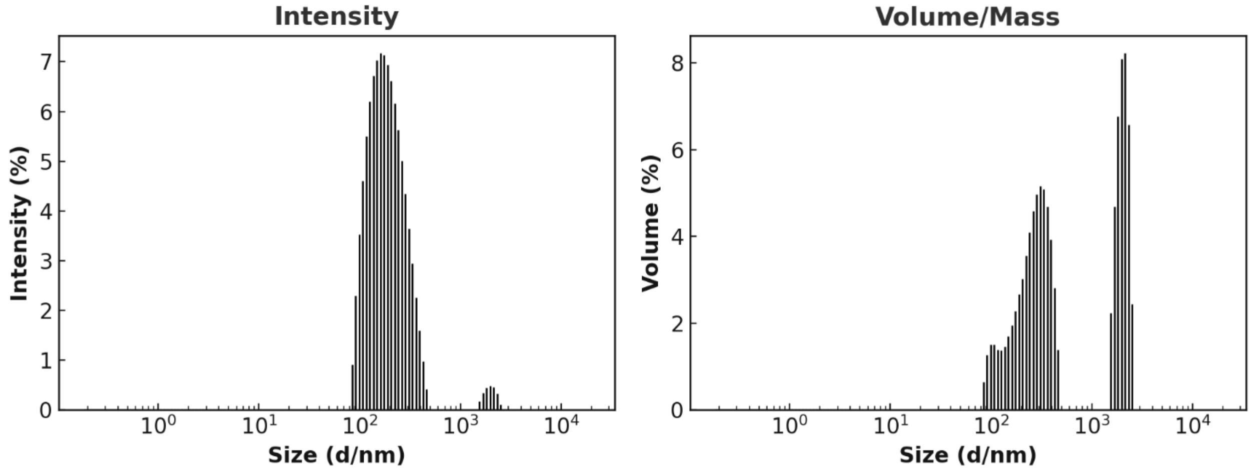

Figure S11. Comparison of DLS-derived intensity and volume/mass distributions for Au@2-propynylamine NPs in deionized water.

Table S7. Peak diameters, intensity percentage (%I) and mass percentage (%M) obtained from DLS measurements of Au@2-propynylamine NPs in DI water.

Peak	Diameter, D (nm)	Intensity percentage (%I)	Mass percentage (%M)
1	283.00	21.6	23.9
2	306.87	18.2	24.5

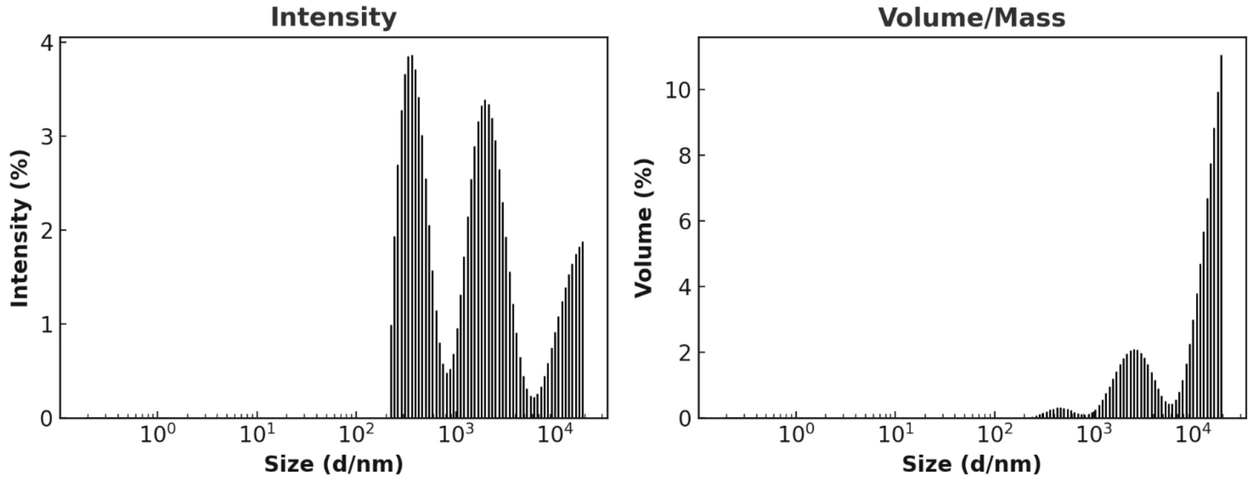

Figure S12. Comparison of DLS-derived intensity and volume/mass distributions for Au@2-propynylamine NPs in PBS.

Table S8. Peak diameters, intensity percentage (%I), and mass percentage (%M) obtained from DLS measurements of Au@2-propynylamine NPs in PBS.

**Peak**	**Diameter, *D* (nm)**	**Intensity percentage (%I)**	**Mass percentage (%M)**
1	586.63	8.1	1.2
2	636.13	6.2	1.0

Table S9. FTIR band assignments for Au@2-propynylamine NPs and Au@(2-propynylamine/Biotin-PEG_4) NPs.

**Wavenumber (cm^{-1})**	**Assignment (functional group)**	**Before (Au@2-propynylamine)**	**After (Au@(…/Biotin-PEG_4))**	**Interpretation**
3300–3400	N–H stretching (primary amine)	Clear band	Slight decrease	Partial coverage by PEG/biotin; overlapping modes
2930, 2870	C–H stretching (aliphatic CH_2/CH_3)	Present	Slight increase	Additional CH_2 groups from PEG backbone
~2100	ν(C≡C) terminal alkyne	Distinct band/shoulder	Attenuated or disappears	Alkyne consumed via CuAAC “click” reaction
1650–1680	Amide I (C=O)	Weak/overlapping	Enhanced	Biotin amide carbonyl introduced
~1540	Amide II (N–H bend + C–N stretch)	Weak/overlapping	Enhanced	Biotin amide vibrations
1450–1350	C–N stretching, CH_2 wagging (triazole + PEG)	Very weak	Clear bands	Triazole ring and PEG contributions
1250–1100	C–O–C stretching (PEG)	Broad, low intensity	Stronger, more defined	PEG ether bands
1050–1000	C–N stretching, C–C skeletal vibrations	Present	Minor shift/intensity change	Local environment modification
<800	Au–N coordination, out-of-plane bending	Largely unchanged	Largely unchanged	Core–ligand framework preserved

***Note**:* Peak positions may shift ±10–20 cm^{-1} depending on baseline and instrument resolution.
